# A conserved sequence motif in the *Escherichia coli* soluble FAD-containing pyridine nucleotide transhydrogenase is important for reaction efficiency

**DOI:** 10.1016/j.jbc.2022.102304

**Published:** 2022-08-04

**Authors:** Michele Partipilo, Guang Yang, Maria Laura Mascotti, Hein J. Wijma, Dirk Jan Slotboom, Marco W. Fraaije

**Affiliations:** 1 Membrane Enzymology Group, Groningen Institute of Biomolecular Sciences & Biotechnology, University of Groningen, Groningen, The Netherlands; 2 Molecular Enzymology Group, Groningen Institute of Biomolecular Sciences & Biotechnology, University of Groningen, Groningen, The Netherlands; 3 IMIBIO-SL CONICET, Facultad de Química Bioquímica y Farmacia, Universidad Nacional de San Luis, San Luis, Argentina

**Keywords:** soluble transhydrogenase, nicotinamide cofactors, flavoprotein, reactive oxygen species, protein engineering, DLS, dynamic light scattering, FDR, flavoprotein disulfide reductase, H_2_O_2_, hydrogen peroxide, KPi, potassium phosphate, MSA, multiple sequence alignment, PMT, photomultiplier, ROS, reactive oxygen species, SEC, size-exclusion chromatography, STH, soluble transhydrogenase

## Abstract

Soluble pyridine nucleotide transhydrogenases (STHs) are flavoenzymes involved in the redox homeostasis of the essential cofactors NAD(H) and NADP(H). They catalyze the reversible transfer of reducing equivalents between the two nicotinamide cofactors. The soluble transhydrogenase from *Escherichia coli* (SthA) has found wide use in both *in vivo* and *in vitro* applications to steer reducing equivalents toward NADPH-requiring reactions. However, mechanistic insight into SthA function is still lacking. In this work, we present a biochemical characterization of SthA, focusing for the first time on the reactivity of the flavoenzyme with molecular oxygen. We report on oxidase activity of SthA that takes place both during transhydrogenation and in the absence of an oxidized nicotinamide cofactor as an electron acceptor. We find that this reaction produces the reactive oxygen species hydrogen peroxide and superoxide anion. Furthermore, we explore the evolutionary significance of the well-conserved CXXXXT motif that distinguishes STHs from the related family of flavoprotein disulfide reductases in which a CXXXXC motif is conserved. Our mutational analysis revealed the cysteine and threonine combination in SthA leads to better coupling efficiency of transhydrogenation and reduced reactive oxygen species release compared to enzyme variants with mutated motifs. These results expand our mechanistic understanding of SthA by highlighting reactivity with molecular oxygen and the importance of the evolutionarily conserved sequence motif.

Pyridine nucleotide transhydrogenases catalyze the transfer of reducing equivalents between the two nicotinamide cofactors NAD(H) and NADP(H) and contribute to cellular redox homeostasis (1, 2). Besides the well-studied membrane transhydrogenases (EC 1.6.1.2) that couple proton transfer across the cell membrane to cofactor transhydrogenation (3), also soluble transhydrogenases (STHs) exist (4). STHs are energy-independent FAD-containing enzymes catalyzing the reversible reaction:

(1) $${\text{NAD}}^{+}+\text{ NADPH }⇌\;\text{NADH }+\;{\text{NADP}}^{+}$$

STHs (EC 1.6.1.1) are evolutionary related to the flavoprotein disulfide reductase (FDR) family (4). The two families share conserved sequence motifs including the GXGXXG motif involved in binding the nucleotide cofactors NAD(P)^+^ and FAD (5) but also show notable differences including a strongly conserved threonine (CXXXXT) in STHs instead of the redox-active disulfide motif CXXXXC in FDRs and a tyrosine replacing histidine in the His-Glu pair (YXXXXE in STHs) in the terminal motif.

The main function of STHs *in vivo* is to oxidize excess of NADPH (6), forming NADH which is thus made available to supply electrons to the respiratory chain (7). As crossroads of the intracellular redox status, STHs from *Escherichia coli* and *Pseudomonas fluorescences* have found wide use in improving the yield of value-added chemicals produced in cell factories. Indeed, by transferring electrons between nicotinamide carriers, transhydrogenases replenish the NAD(P)H pool required by the metabolic pathway of the target compound. However, most metabolic engineering reports employing STHs benefit from the NADPH generation at the expense of NADH oxidation (8, 9, 10, 11), a reaction that corresponds to the opposite direction proposed as native function of STHs (Equation 1). Similarly to their use in metabolic engineering, the emerging field of cell-free synthetic biology (12) (sometimes referred to as “synthetic biochemistry” (13)) has also shown the value of STHs to sustain the synthesis of diverse classes of biomolecules, from opioids as hydromorphone (14) to fatty acids derivatives as in the case of p-nitrophenoxydecanoic acid (15), up to the formation of antioxidant species such as reduced glutathione within phospholipidic compartments (16). These last two examples exploit the NADPH-generating action of the soluble transhydrogenase from *E. coli*, catalyzing the supply of demanded reactants. The above highlights the reversible nature of the enzymatic transhydrogenation rendering transhydrogenases attractive and flexible biocatalysts.

Despite a partial biochemical characterization (17) of the soluble transhydrogenase SthA (also known as UdhA) from *E. coli*, numerous mechanistic aspects remain to be elucidated. Not only is the mechanism by which NADPH is formed still obscure but also possible unwanted side reactions of the flavoprotein with molecular oxygen have not been studied (18, 19, 20). Insight into these properties of SthA would facilitate its broader use in metabolic engineering and industrial biomanufacturing.

In this work, we present the study of the purified soluble transhydrogenase (SthA) from *E. coli*. We initially characterize the reaction in which (thio)NADPH is produced from NADH. Then, we describe a hitherto unreported reactivity of SthA with molecular oxygen in the absence of oxidized cofactors as electron acceptors. This reaction involves full reduction of FAD and it leads to the formation of superoxide anion and hydrogen peroxide (H_2_O_2_). The reactivity of the flavoprotein with dioxygen and production of reactive oxygen species (ROS) also takes place during transhydrogenation in the presence of the oxidized cofactor (thio)NADP^+^ as electron acceptor, although only as minor side-reaction (2%) compared to the hydride transfer between nicotinamide cofactors. Finally, we explore the characteristic CXXXXT motif in the FAD-binding domain of SthA, both by replacing cysteine (C45A) and by restoring the redox-active disulfide center typical of FDRs *via* threonine mutagenesis (T50C). We determined the ability of C45A and T50C to bind FAD and to perform transhydrogenation, as well as to produce ROS. These SthA mutants reveal that the conservation of the CXXXXT motif in STHs is necessary for efficient enzymatic transhydrogenation.

## Results

### The purified SthA from *E. coli* is an octameric FAD-containing protein

**Figure 1:**
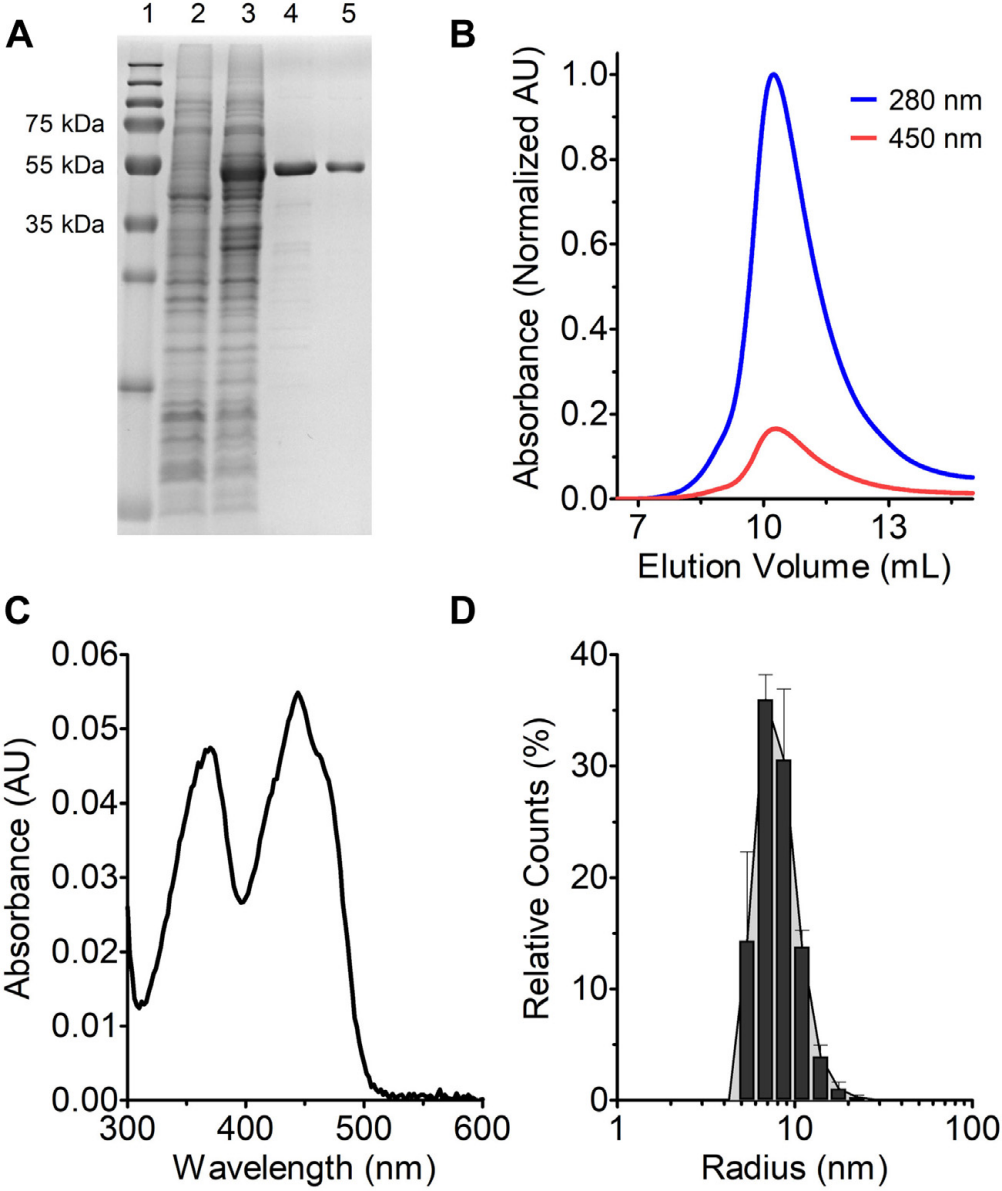
**Purification and properties of the flavoenzyme soluble transhydrogenase from *Escherichia coli*.***A*, SDS-PAGE analysis of SthA purification. Lane 1, protein markers; lane 2, extract of noninduced cells; lane 3, extract of induced cells; lane 4, affinity chromatography fraction, lane 5, size-exclusion chromatography (SEC) fraction. *B*, size-exclusion chromatogram of purified SthA. The protein elution on a Superdex 200 10/300 column was monitored at 280 nm (*blue line*) and 450 nm (*red line*). *C*, absorbance spectrum of 4.0 μM SthA. *D*, dynamic light scattering (DLS) of the purified enzyme. The estimated radius is 8.2 ± 0.2 nm, with a molecular weight of 467.3 ± 23.0 kDa, and polydispersity of 29.1 ± 7.6%. The DLS profile is the result of three different purification batches (n = 3), while the errors as SDs are shown only above for clarity. STH, soluble transhydrogenase.

SthA from *E. coli* K-12 with a 10x-histidine tag at the C-terminus was overproduced in *E. coli* MC 1061 (Fig. 1*A*). The yellow protein was purified from the soluble fraction by affinity chromatography followed by size-exclusion chromatography (SEC profile shown in Fig. 1*B*). The absorbance spectrum of the purified protein indicated the presence of bound oxidized FAD with absorbance maxima at 370 nm and 450 nm (Fig. 1*C*). For accurate determination of enzyme concentration (21), the extinction coefficient at 450 nm was determined: ε_450_ = 12.1 mM^−1^ cm^−1^. The protein retained its intense yellow color during the purification protocol suggesting a tightly bound FAD cofactor. This was confirmed by measuring the ratio between the wavelengths at 280 and 450 nm (A_280/450_): a value of around 6 indicated that most protein is in its holo form, although we could detect some small loss of FAD upon SEC (Fig. S1). The protein had a tendency to aggregate upon storage at -80 °C, which was prevented by the addition of glycerol (5–10%).

SthA protomers have a calculated molar mass of around 54 kDa as confirmed by SDS-PAGE analysis (Fig. 1*A*). Yet, the hydrodynamic radius of 8.2 ± 0.2 nm measured by dynamic light scattering (DLS) indicated a molar mass of 467.3 ± 23.0 kDa (Fig. 1*D*), suggesting an octameric native state. This result was confirmed by interpolating the elution volume upon SEC using standards of known molecular weight (Fig. S2), obtaining a molar mass of 437.7 ± 13.9 kDa. Electron microscopy of the negatively stained–purified SthA (Fig. S3) showed particles with a size matching the hydrodynamic radius obtained by DLS.

### Characterization of the NADH-consuming reaction

**Figure 2:**
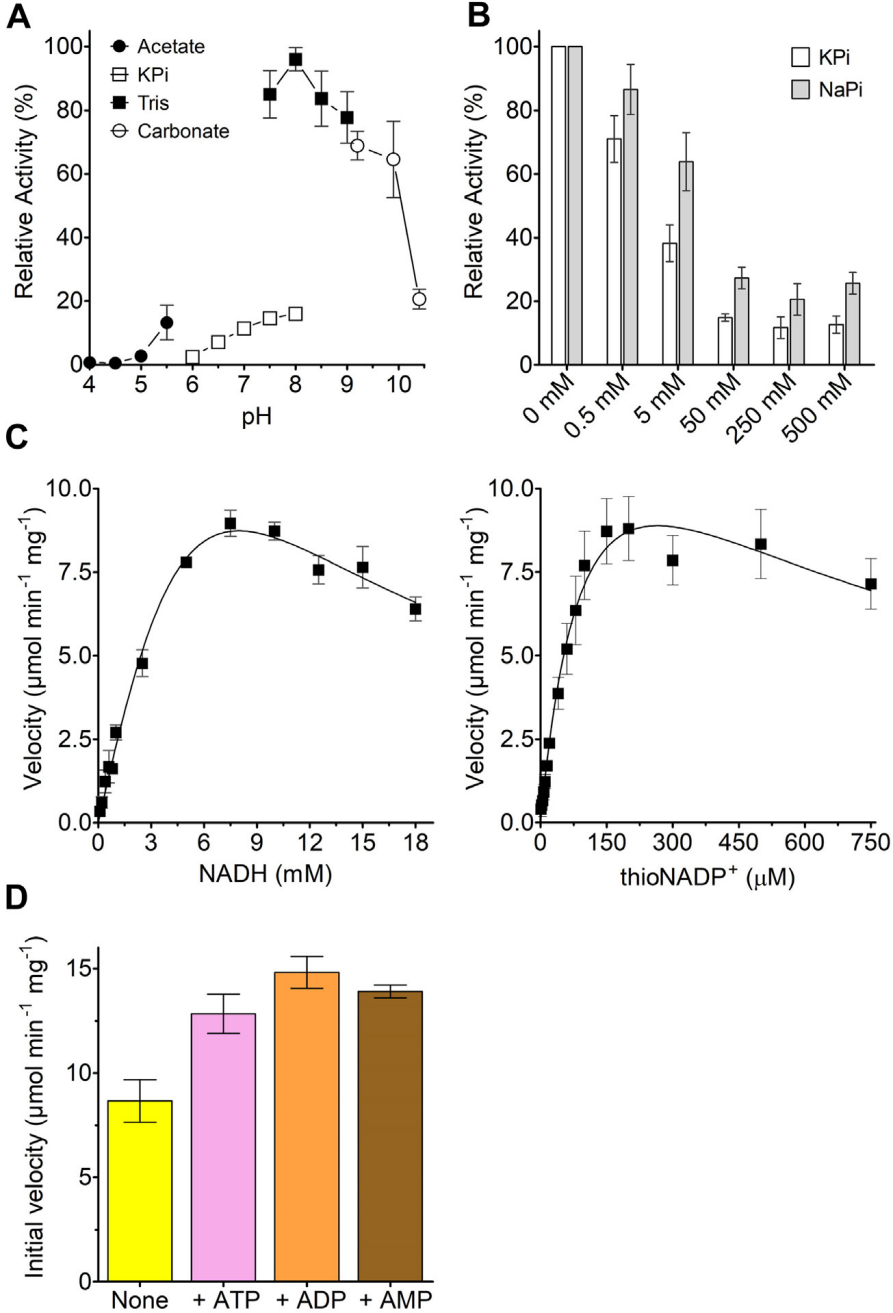
**SthA activity and kinetics.***A*, buffer and pH optimum of the thioNADPH formation mediated by SthA. The enzymatic activity of 20 nM SthA was measured in the presence of 0.5 mM NADH and 0.1 mM thioNADP+ at different pH values, using 100 mM buffers (legend in the figure). The activity in 100 mM Tris at pH 8.0 was set as 100%. The data comes from biological quadruplicates (n = 4), illustrating the s.e.m. as error bars. *B*, effect of phosphate on the SthA-transhydrogenation. Increasing the amount of phosphate in the form of potassium phosphate (KPi as *white bars*) or sodium phosphate (NaPi as *gray bars*), we observed a decrease in the reaction rate that leveled off around 50 mM phosphate. 100% activity was fixed as the transhydrogenation rate reached at 30 °C in the reaction mixture devoid of phosphate and composed of 50 mM Tris at pH 7.5 (Activity buffer), 20 nM SthA, 0.15 mM thioNADP+, and 1.0 mM NADH (n = 3, error bars display the s.e.m.). *C*, observed rate of thioNADP+ reduction by SthA as function of the concentrations of the substrates NADH and thioNADP+ at 30 °C. The assays were carried out in 50 mM Tris, pH 7.5. The kinetics data in the graphs were obtained from three independent replicates (n = 3), while the error bars represent the s.e.m. *D*, catalytic activation in the presence of the adenine nucleotides. The reaction conditions in the absence of any activator (*yellow bar*) were 50 mM Tris at pH 7.5, 1.0 mM NADH, 0.15 mM thioNADP+, 20 nM SthA. The effect of ATP (*pink bar*), ADP (*orange bar*), or AMP (*brown bar*) was evaluated using the same reaction mixture composition, by including 5.0 mM of each nucleotide. The data comes from three independent repetitions (n = 3, the error bars illustrate the s.e.m.). STH, soluble transhydrogenase.

Although there are several studies demonstrating the SthA capacity to mediate the hydride transfer from NADH to NADP^+^ (8, 9, 10, 11, 15, 16), a thorough kinetic study of SthA is still lacking. First, we investigated which buffer and pH conditions are optimal for the hydride transfer from NADH to thioNADP^+^ (Fig. 2*A*) by following the reduction of the latter. The replacement of the oxygen with a sulfur in the amide side-chain of the nicotinamide moiety of thioNADP^+^ allows to monitor the cofactor reduction at 400 nm, discriminating it from NAD(P)H which instead absorbs at 340 nm (22). The optimal buffer for (thio)NADPH generation was 100 mM Tris, tested in the pH range of 7.5 to 9.0, showing the highest SthA catalytic performance at pH 8.0. In potassium phosphate (KPi) buffer, the activity of SthA was strongly reduced. Very low activity (∼2% when compared with Tris, pH 8.0) was detected in KPi at pH 6.0. At pH values of 7.5 and 8.0, the activity in KPi buffer was over 6-fold lower than in Tris buffer of the same pH, indicative of inhibition by phosphate (Fig. 2*B*). By increasing the KPi concentration, we observed that the SthA-transhydrogenation activity leveled off to a basal activity of 15% at 50 mM or higher. When potassium was replaced with sodium phosphate, we observed the same inhibitory trend but with slightly higher basal activity values (20–25%) than those of KPi. The low activity was ascribed exclusively to the presence of phosphate since we did not detect any effect on the SthA-mediated transhydrogenation at high ionic strength with sodium or potassium chloride (Fig. S4).

Then, we determined the kinetic parameters (Fig. 2*C* and Table 1) of the NADH-consuming reaction catalyzed by SthA. Fixing the amount of thioNADP^+^ at 0.5 mM, we calculated an apparent affinity constant (*K*_M_) for NADH (Fig. 2*C*, on the left) of 2.6 ± 0.4 mM, with a turnover number (*k*_CAT_) of 9.2 ± 0.5 s^−1^. At high NADH concentrations (above 7 mM), substrate inhibition was observed, with an estimated inhibition constant (*K*_I_) of 12.1 ± 2.0 mM. In the case of thioNADP^+^ (Fig. 2*C*, on the right), we estimated a significantly lower *K*_M_ (121 ± 40 μM) than that of NADH (kept constant at 10.0 mM during the kinetics), while the *k*_CAT_ value was similar (15.3 ± 0.5 s^−1^) to the one calculated for the reduced cofactor. Substrate inhibition was again evident at high concentrations of thioNADP^+^, resulting in a *K*_I_ value of 585 ± 257 μM.

**Table 1:** Kinetic parameters of SthA for the transhydrogenase activity (upper panel) and oxidase activity (lower panel)

Transhydrogenase activity: NADH + thioNADP^+^ → NAD^+^ + thioNADPH						
Substrate	*K*_M_ (mM)	*K*_I_ (mM)	*V*_MAX_ (μmolˑ min^−1^ˑmg^1^)	*k*_CAT_ (s^−1^)	*k*_CAT_/*K*_M_ (s^−1^ˑmM^−1^)	Cosubstrate
NADH	2.6 ± 0.4	12.1 ± 2.0	10.2 ± 0.5	9.2 ± 0.5	3.6	0.5 mM thioNADP^+^
thioNADP^+^	0.12 ± 0.04	0.59 ± 0.26	17.0 ± 3.5	15.3 ± 3.1	127.5	10.0 mM NADH

**Oxidase activity: NAD(P)H + O_2_ → NAD(P)^+^ + H_2_O_2_ + O_2_^•-^**						

**Substrate**	**Apparent *K*_M_ (μM)**	***K*_I_ (mM)**	**Apparent *V*_MAX_ (nmolˑ min^−1^ˑmg^1^)**	**Apparent *k*_CAT_ (s^−1^)**	***k*_CAT_/*K*_M_ (s^−1^ˑmM^−1^)**	**Cosubstrate**

NADH	49.1 ± 10.4	--	137.1 ± 8.8	0.12 ± 0.01	2.4	0.2 mM O_2_ (53)
NADPH	91.8 ± 30.5	--	221.0 ± 28.3	0.20 ± 0.03	2.2	0.2 mM O_2_

The measurements were carried out in 50 mM Tris, pH 7.5 at 30 °C in biological triplicate (*n* = 3, the errors indicate the s.e.m.), using for each of them single technical replicates.

Since the nucleotide adenine cofactors AMP, ADP, and ATP are reported as activators of SthA for the reaction in the opposite direction (production of NADH) (17), we investigated whether they also affected the reaction generating (thio)NADPH (Fig. 2*D*). Indeed, at the concentration of 5.0 mM, all three adenine nucleotides increased the rate of thioNADPH formation (reported as percentages in Table S1). ADP was the best reaction activator, followed by AMP and ATP, respectively.

### The uncoupling activity: SthA acting as NAD(P)H oxidase

**Figure 3:**
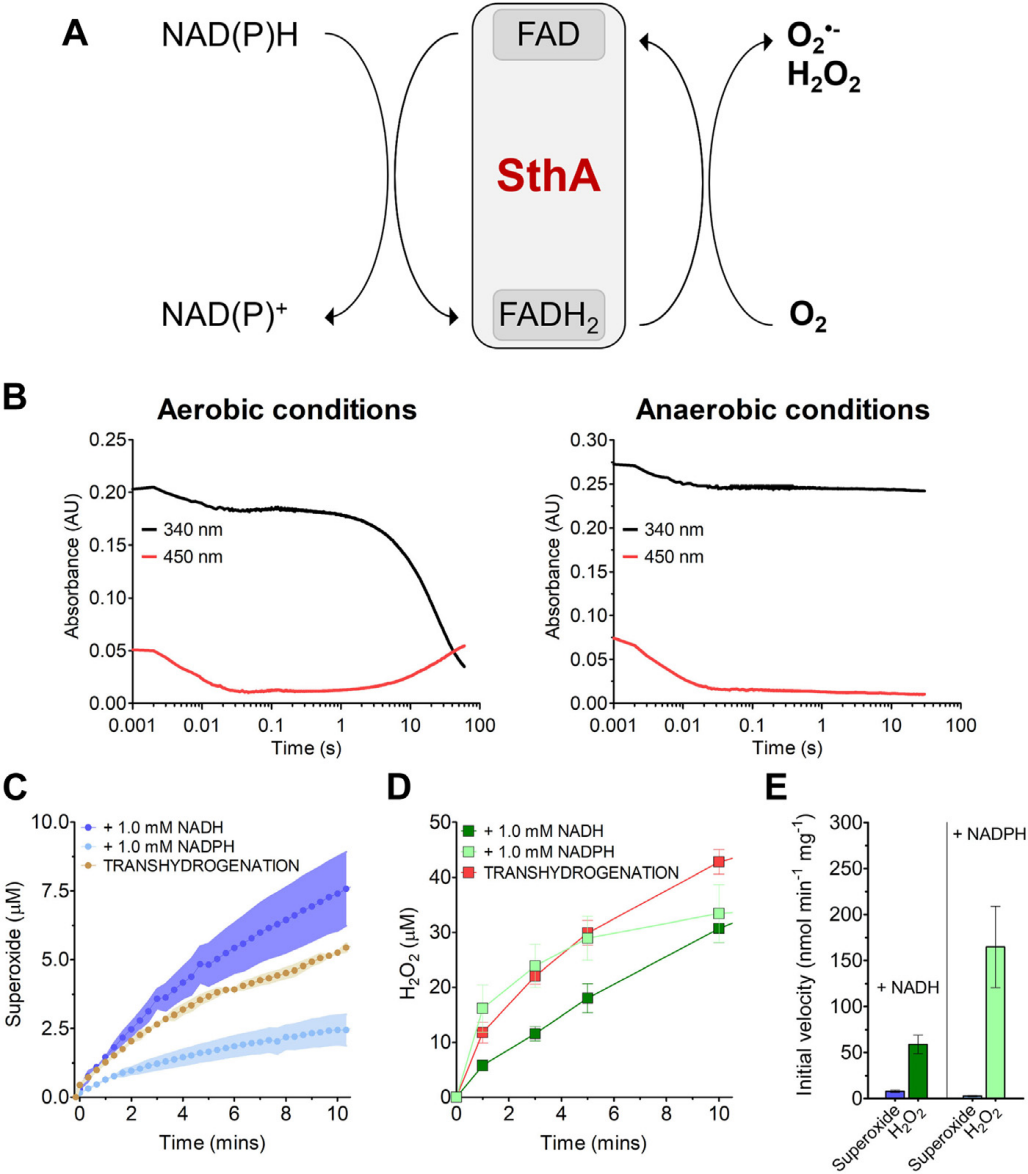
**The uncoupled oxidase activity of SthA.***A*, scheme reaction of the uncoupling activity oxidizing NAD(P)H in the presence of dioxygen but without NAD(P)+ as electron acceptor. The reducing equivalents are initially transferred to the flavoprotein and then to molecular oxygen, generating hydrogen peroxide and superoxide anion. *B*, oxygen-dependent NADH oxidation mediated by SthA in the absence of NAD(P)+ as electron acceptor. In aerobic conditions (*left panel*), the transient reduction of the embedded FAD (*red line*) allows the oxidation of NADH into NAD+ (*black line*), with oxygen as final electron acceptor. Carrying out the same reaction anaerobically (*right panel*), the flavin is reduced into FADH2 and it retains the reducing equivalents without being reoxidized at the expense of O2. Both the reactions were triggered by mixing 50 μM NADH and 7.5 μM (*left*) or 10.0 μM (*right*) SthA in buffer 50 mM Tris at pH 7.5, NaCl 0.15 M, glycerol 5%. *C*, superoxide detection from the SthA-mediated uncoupling and transhydrogenase activities. The uncoupling oxidase activity is followed with 1.0 mM NADH (*dark blue line*) or 1.0 mM NADPH (*light blue line*). Also, the transhydrogenation between 1.0 mM NADH and 0.2 mM thioNADP+ (*ocher line*) leads to superoxide generation. All the reactions were carried out in biological triplicate, each of them with a single technical replicate (n = 3, s.e.m. represented as error bars), at 30 °C in 50 mM Tris at pH 7.5, 20 μM ferricytochrome c, and started by adding 600 nM SthA. *D*, hydrogen peroxide detection from the SthA-mediated uncoupling and transhydrogenase activities. Using the same reaction conditions employed for the superoxide measurements in Fig.3*C*, we followed the uncoupling activity in the presence of NADH (*dark green squares*) or NADPH (*light green squares*) at different time points of the reaction. H2O2 was also formed as the result of the transhydrogenation (*red squares*). The data come from independent triplicates using single technical replicates (n = 3, the error bars show the s.e.m.), while the amount of produced H2O2 was quantified for each time point in xylenol assay solution (125 μM xylenol orange, 100 mM D-sorbitol, 250 μM (NH4)2Fe(SO4)2, 25 mM H2SO4), upon incubation for 15 min at 30 °C. *E*, reaction rates of the ROS formation from the uncoupling activity with NADH and NADPH. The initial velocity values refer to the first minute of the reactions reported in Figure 3, *C* and *D*. The *blue bars* show the superoxide production (in *dark blue* starting from 1.0 mM NADH, in *light blue* from 1.0 mM NADPH), and the *green bars* illustrate the formed hydrogen peroxide from equimolar NADH (*dark green*) and NADPH (*light green*). STH, soluble transhydrogenase; ROS, reactive oxygen species.

In recent years, a growing number of reports in the literature have shown how various flavoenzymes display side reactivity with molecular oxygen, forming ROS (19, 23). We decided to test if SthA can also catalyze the formation of ROS. We hypothesized that this uncoupling activity (Fig. 3*A*) would start with the reduction of FAD into FADH_2_ by NAD(P)H and then be followed by the reoxidation of the reduced flavoenzyme by dioxygen. Such transfer of reducing equivalents to the final donor (O_2_) would lead to the formation of the ROS species superoxide anion and/or H_2_O_2_. We tested this hypothesis by the addition of NADH to a reaction mixture containing SthA and devoid of any oxidized cofactor NAD(P)^+^ with or without oxygen (Fig. 3, *B* and *C*). By monitoring the absorbance at 340 and 450 nm with a stopped-flow setup (full absorbance spectra available on Fig. S5), we followed at the same time the redox status of both the nicotinamide and the flavin-embedded cofactors. Using the stopped-flow instrument, we could not only monitor rapid kinetics but it also allowed to control the oxygen concentration in the reactions (24, 25). In aerobic conditions (Fig. 3*B*, left panel), a rapid reduction of the prosthetic flavin (≤30 ms) was initially observed—as absorbance decrease at the wavelength of 450 nm (red line)—with the concomitant oxidation of a fraction of the NADH pool visible at 340 nm (black line). Following a short stationary phase, the formed FADH_2_ and the remaining NADH were fully oxidized in the range of tens of seconds. Under anaerobic conditions (Fig. 3*B*, right panel), the first half-reaction was identical to the aerobic condition. However, the second half-reaction did not take place and FADH_2_ remained in the reduced state, since no O_2_ was available to accept the electrons taken over by the flavin. Consequently, also NADH could not be further oxidized to NAD^+^.

We then determined the kinetic parameters for the oxidative activity using either NADPH or NADH as electron donor in the absence of any NAD(P)^+^. This revealed that SthA aerobically oxidizes both NADH and NADPH (Table 1 and Fig. S6 for kinetic plots), essentially acting as an NAD(P)H oxidase with a *k*_CAT_ of 0.1 to 0.2 s^−1^. For this uncoupling reaction, it shows a higher affinity for NADH than NADPH but a higher turnover number when NADPH was electron donor. Using NADH, SthA showed a 75-fold higher *k*_CAT_ value for the transhydrogenation than the oxidase activity, although the catalytic efficiency (*k*_CAT_/*K*_M_) for the hydride transfer between cofactors was only 1.5 times higher than the oxygen reduction. The millimolar range of the *K*_M_ for NADH during transhydrogenation lies behind such a low catalytic efficiency.

Next, we focused on the formation of the ROS species. We used ferricytochrome *c* (26) as the reporter for production of superoxide anion. Time course experiments revealed that SthA produces superoxide anion in the order of a few micromolar (Fig. 3*C*), in the presence of an excess of 1.0 mM NAD(P)H as substrate. After 10 min of reaction, NADH led to the accumulation of almost 7.5 μM of superoxide anion (dark blue line), three times higher than the superoxide reached from an equimolar amount of NADPH (light blue line). Intriguingly, we also detected superoxide formation during the transhydrogenation between 1.0 mM NADH and 0.15 mM thioNADP^+^ (ocher line), which generated similar levels of radical species to those observed in the absence of oxidized nicotinamide cofactors as electron acceptors. Then, we tested both the uncoupling and the transhydrogenase activities under the same experimental conditions as in Figure 3*C* but this time by measuring the production of H_2_O_2_ (Fig. 3*D*). H_2_O_2_ was produced in all cases, at higher levels than the superoxide anion (30–40 μM H_2_O_2_ in 10 min of reaction), showing that H_2_O_2_ is the main product of the SthA uncoupling activity, which was confirmed by comparing the ROS formation reaction rates (Fig. 3*E*). Indeed, we estimated the generation of H_2_O_2_ (green bars) to be more than 5 times and 30 times faster than the superoxide anion (blue bars), respectively from the oxidation of NADH and NADPH. As expected, the reaction rates for the ROS release fit well with the turnover numbers calculated for the NAD(P)H consumption (Table 1).

### Investigating the role of conserved residues in the CXXXXT motif

**Figure 4:**
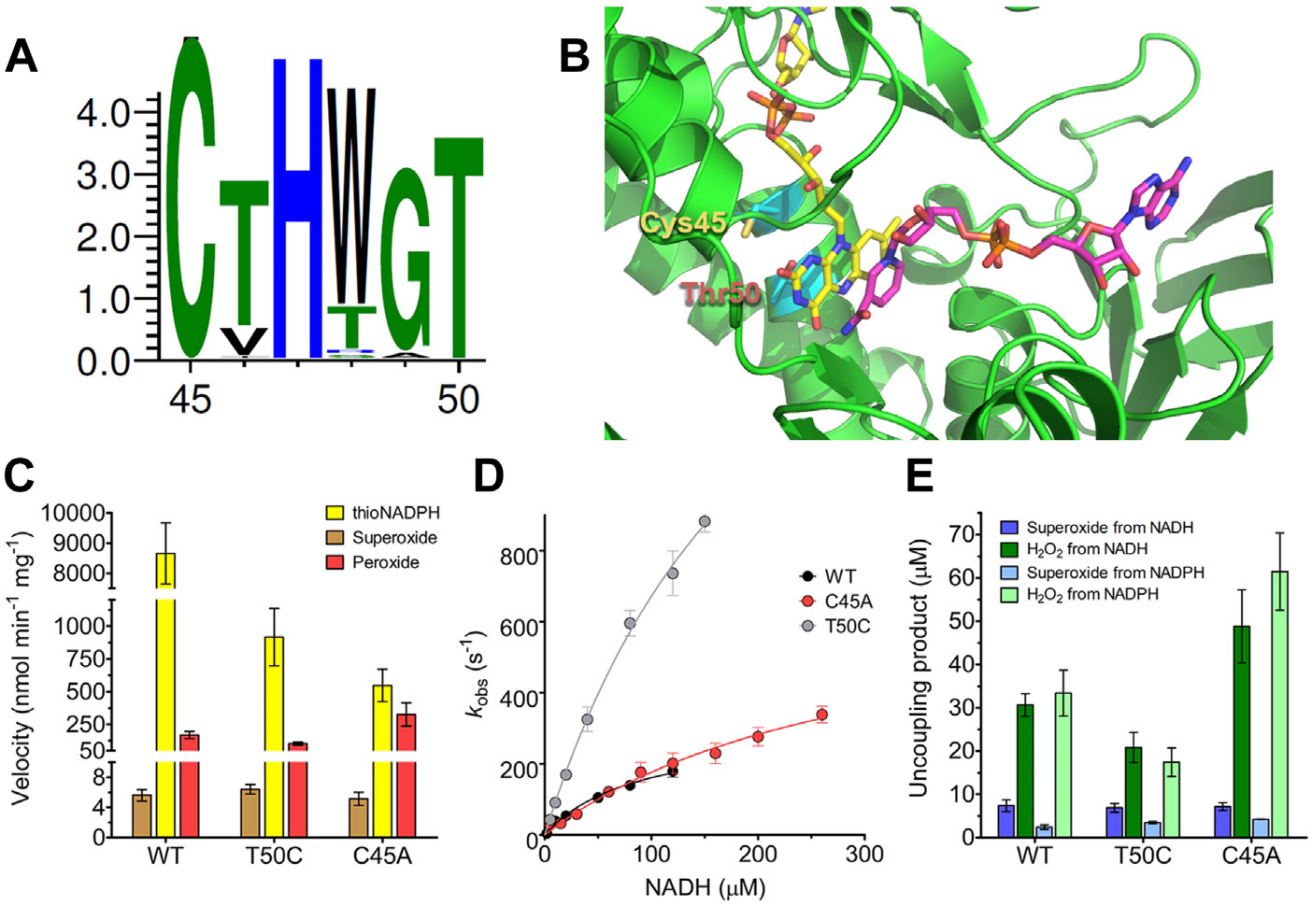
**The role of the conserved CXXXXT motif in SthA.***A*, a multiple sequence alignment containing STHs was built to obtain a sequence logo with the server WebLogo3 (28). Amino acids are colored according to their chemistry (polar in *green*, basic in *blue*, and hydrophobic in *black*). *B*, Alphafold-predicted structure of SthA. The FAD and NAD+ cofactors were docked based on the superimposition of the crystallographic structure of the bacterial lipoamide dehydrogenase from *Thermus thermophilus* HB8 (PDB entry:2EQ7). *C*, comparison of the initial rates of thioNADPH, superoxide, and hydrogen peroxide formation during the transhydrogenase activity of the SthA-variants. The transhydrogenation between 1.0 mM NADH and 0.15 mM thioNADP+ leads to the generation of thioNADPH (*yellow bars*), hydrogen peroxide (*red bars*), and superoxide anion (*ocher bar*) at different rates. The final rates represent the average of independent triplicates (n = 3), the errors report the s.e.m.. *D*, pre–steady-state kinetics for the reductive half-reaction of SthA mutants. The values of the reduction rate constant (kred) of the SthA variants and apparent dissociation of NADH constant (Kd) come from biological triplicates (n = 3, error bars display the s.e.m.). *E*, H2O2 and superoxide production from the uncoupling activity of the SthA-variants. A similar superoxide release from the oxidase activity is observed when NADH (*dark blue bar*) or NADPH (*light blue bar*) are cosubstrates. The peroxide formation displayed more significant differences among the variants, both from NADH (*dark green bar*) and NADPH (*light green bar*). The amount of uncoupled products is calculated from biological triplicates, each of them with a single technical replicate (n = 3, the error bars represent the s.e.m.), after 10 min of reaction at 30 °C including 50 mM Tris at pH 7.5, 1.0 mM NADH or NADPH, 600 nM SthA. The incubation in xylenol assay solution was carried out in the dark at at 30 °C for 15 min. STH, soluble transhydrogenase.

Unlike the sequence-related FDR family, STHs characteristically contain a threonine replacing the C-terminal cysteine in the CXXXXT motif equivalent to the redox-active disulfide center in FDRs. Due to its evolutionary conservation among STHs (Figs. 4*A*, and S7 for the phylogenetic tree), the presence of the threonine in position 50 suggests a role for this residue to operate the enzymatic transhydrogenation. On the other hand, the cysteine in position 45 emerges as the link between STH and FDR families, as it is mechanistically crucial for the reduction of thiol substrates in the latter protein group (4). Therefore, we characterized the single-mutants C45A and T50C to establish the importance of Cys45 and Thr50 for the catalytic properties of SthA.

Compared to the WT enzyme, both the mutants revealed a lower flavin content after the protein purification (Table 2 and Fig. S8 for the SEC profiles), indicating that Cys45 and Thr50 might promote a more stable embedding of the prosthetic FAD cofactor in SthA. Indeed, *in silico* docking of the FAD molecule to the Alphafold-predicted structure of SthA (Fig. 4*B*) revealed the close proximity of Cys45 and Thr50 to the ribityl moiety and the N5 and N10 atoms of the isoalloxazine ring, respectively. The lower capacity to retain the prosthetic group in the SthA mutants even matched the amount of unbound FAD eluted during the purification procedure (Figs. S8 and S1). When we verified their capacity to transfer reducing equivalents from NADH to thioNADP^+^, both C45A and T50C displayed a comparable one-order-of-magnitude loss of the activity found in the WT protein (Fig. 4*C*, yellow bars).

**Table 2:** Flavin content of the SthA-variants designed in our study

Variant	Ratio 280/450	*ε*_450 nm_ (mM^-1^ cm^-1^)
WT	6.0 ± 0.3	12.1
C45A	7.4 ± 0.2	13.0
T50C	8.8 ± 0.2	12.6

The ratio 280/450 corresponds to the ratio between the wavelengths at 280 and 450 nm measured during the elution of the purified protein sample on SEC. The reported values correspond to the average of five different purification batches (*n* = 5, s.e.m. as errors). The extinction coefficients at 450 nm (*ε*_450 nm_) for each protein variant were determined according to established protocols for flavoproteins (21).

We then studied the reductive half-reaction by determining the apparent dissociation constant (*K*_d_) for NADH and reduction rate constant (*k*_red_) of the protein-embedded flavin by stopped-flow kinetic measurements (Fig. 4*D* and Table 3, representative spectra available in Fig. S9). WT SthA was found to be efficiently and fully reduced by NADH with a *k*_red_ of 303 s^−1^ and *K*_d_ of 88 μM. Mutations in the CXXXXT motif increased the *K*_d_ by 3 to 4 fold compared to WT SthA. The higher *K*_d_ values suggest that the combination of Cys45 and Thr50 is needed for optimal binding of the nicotinamide substrate. On the other side, both mutant SthAs displayed significantly higher *k*_red_ values. The C45A mutation doubled the *k*_red_, while restoration of the disulfide center through the T50C mutation led to a 7-fold increase of the reduction rate.

**Table 3:** Pre–steady-state kinetic parameters for the reductive half-reaction of SthA mutants

Variant	Substrate	*k*_red_ (s^-1^)	*K*_d_ (μM)
WT	NADH	303 ± 50	88 ± 27
C45A	NADH	710 ± 160	300 ± 100
T50C	NADH	2200 ± 400	227 ± 61

The values of reduction rate constant of the SthA variants (*k*_red_) and apparent dissociation of NADH constant (*K*_d_) come from biological triplicates (*n* = 3, s.e.m. as errors) measured by stopped-flow spectrophotometry in anaerobic conditions.

Then, we tested if the mutations of the CXXXXT motif affected the production of ROS species. We compared ROS formation after 10 min of reaction starting from 1.0 mM NADH or NADPH (Fig. 4*E*). Overall, the amount of superoxide anion (blue bars) showed no major differences between the mutants and the WT protein, resulting in approximately 7 μM of produced superoxide by oxidizing NADH and no more than 4 μM of superoxide from NADPH in all the SthA constructs. We observed larger differences in H_2_O_2_ production (green bars) between WT and SthA mutants. The WT enzyme generated around 30 μM H_2_O_2_ by both oxidizing NADH and NADPH. T50C proved to be the least efficient variant in releasing peroxide (20 μM H_2_O_2_ with NADH and 17 μM H_2_O_2_ with NADPH). Intriguingly, the replacement of Cys45 with alanine made the protein more prone to form peroxide as a consequence of the uncoupling activity, leading to 1.5 and 2 times the H_2_O_2_ produced by WT with NADH and NADPH as cosubstrates, respectively.

Since the ROS release does not occur exclusively in the absence of oxidized nicotinamide cofactors, but also during the transhydrogenase activity (see Fig. 3, *C* and *D*), we determined the initial rates of product formation during the hydride transfer from NADH to thioNADP^+^ (Fig. 4*C*). Using the values obtained from each variant to generate thioNADPH, H_2_O_2_ and superoxide anion (the sum of the correspondent initial velocities was considered as 100% efficiency) and assuming no other product is formed, we calculated the respective coupling efficiencies (Table 4), expressed as the percentage of the reducing equivalents of NADH consumed to form products during the transhydrogenation.

**Table 4:** The coupling efficiency values of the SthA variants WT, C45A, and T50C

Variant	Maximum Efficiency	Coupling efficiency (%)		
		thioNADPH	Peroxide	Superoxide
WT	a	98.0 ± 9.4	1.9 ± 0.3	0.1 ± 0.0
C45A	a b	6.2 ± 1.1 62.3 ± 11.5	3.7 ± 0.8 37.1 ± 8.2	0.1 ± 0.0 0.6 ± 0.1
T50C	a c	10.4 ± 2.0 89.2 ± 17.4	1.2 ± 0.1 10.2 ± 0.9	0.1 ± 0.0 0.6 ± 0.1

The reported values were obtained from Figure 4, C, by converting the numbers corresponding to the initial velocities in percentages. The errors report the s.e.m. of independent triplicates (*n* = 3).

a, The sum of the initial velocities calculated for WT SthA-producing thioNADPH, H_2_O_2_, and superoxide was set as 100%.

b, The sum of the initial velocities calculated for C45A SthA-producing thioNADPH, H_2_O_2_, and superoxide was set as 100%.

c, The sum of the initial velocities calculated for T50C SthA-producing thioNADPH, H_2_O_2_, and superoxide was set as 100%.

WT SthA showed the highest coupling efficiency, as it channels 98% of the NADH hydrides to (thio)NADP^+^; only 2% of the reducing equivalents are transferred to the molecular oxygen, which is mostly converted into H_2_O_2_. The restoration of the disulfide center (T50C) compromised the activity of the transhydrogenase by more than 10 fold compared to the WT, but also in this case, the majority of NADH electrons (89%) are mostly destined for transhydrogenation. Nonetheless, the relative fluxes toward the peroxide and superoxide are 5 to 6 times higher than in the WT. The removal of cysteine from the CXXXXT motif (C45A) also led to a sharp decline in coupling efficiency with only 62% of the reducing equivalents of NADH being transferred to (thio)NADP^+^, while the remaining pool of hydrides is intercepted by the dioxygen in the final form of 37% H_2_O_2_ and a negligible 1% superoxide anion.

## Discussion

SthA from *E. coli* encompasses several properties found in other STHs characterized to date, urging its study as a prototypical enzyme for this protein family. Common to all STHs, it is a flavoprotein-containing FAD, whose transhydrogenase activity is optimal between pH 7.5 and 8.0, inhibited by high concentrations of phosphate (27, 28), and affected by the presence of the nucleotide adenines ATP, AMP, and ADP (4, 22). In addition to this, SthA contains the typifying CXXXXT motif that sets the STHs apart from the evolutionary related family of FDRs. In the latter enzymes, a CXXXXC motif is present that contains the two catalytically active cysteines. Only a few bacterial transhydrogenases have been studied in some detail. This has shown that they typically form oligomers. SthA differs in oligomeric assembly from the soluble transhydrogenases of *Azotobacter vinelandii* and *Pseudomonas fluorescens*. While the latter two transhydrogenases organize themselves into filamentous structures of hundreds of nanometers long and slightly more than 10 nm wide (29, 30), the *E. coli* ortholog forms octamers with a molar mass of around 440 kDa (Figs. 1*D*, S2 and S3). Experiments performed by Boonstra *et al*. (29) on the chimerization of the aforementioned STHs suggested that the C-terminal domain (last 113 amino acids) of *A. vinelandii* and *P. fluorescens* SthAs plays an important role for the supramolecular structure of these homologs. In fact, when the C-terminal part belonging to SthA of *A. vinelandii* was replaced with the C-terminus of the enzyme of *E. coli*, the filamentous assembly was lost.

The octameric SthA could be well expressed and purified as recombinant protein, allowing a detailed biochemical study. In this work, we focused on unraveling the catalytic properties of SthA. For monitoring transhydrogenase activity, the nicotinamide cofactor mimic thioNADP^+^ was used as surrogate substrate to follow hydride transfer from NADH to thioNADP^+^. It was found that Tris buffer at slightly alkaline pH values is the best buffer for optimal transhydrogenation activity (Fig. 2*B*). A detrimental effect of high phosphate concentrations on activity was also uncovered (Fig. 2*C*), although complete inhibition of SthA was never achieved even in the presence of extreme high phosphate concentrations. The inhibitory effect may be caused by competition of phosphate and the nicotinamide cofactors, containing phosphate moieties, for the same binding pocket.

We report for the first time a further novel catalytic activity mediated by SthA: NAD(P)H oxidase activity. We have analyzed the ability of SthA to utilize NAD(P)H and dioxygen using steady state and pre–steady state kinetic measurements. The kinetic mechanism of SthA can be dissected into two half-reactions. In the reductive half-reaction, a hydride is transferred from the reduced nicotinamide to the flavin cofactor, resulting in a fully reduced protein-bound flavin cofactor (FADH_2_). This is followed by the second half-reaction in which FAD is reoxidized using a suitable (nicotinamide) electron acceptor. For transhydrogenase activity, it would be an oxidized nicotinamide cofactor. We investigated whether SthA is also able to use dioxygen as alternative electron acceptor, thus leading to the formation of ROS (Fig. 3, *A* and *B*). It was found that SthA can indeed function as a NAD(P)H oxidase. By comparing the kinetic parameters of the oxidase and transhydrogenase activities for NADH (Table 1), we found that transhydrogenation (*k*_CAT_ = 9.2 s^−1^) is significantly faster than the oxidase activity (*k*_CAT_ = 0.1 s^−1^). The determination of the products from the oxidase activity (Fig. 3*E*) established H_2_O_2_ as the main uncoupled reaction product, although a small fraction of the electrons transferred to dioxygen generate the superoxide anion. As expected, the turnover numbers calculated for the NAD(P)H consumption (Table 1) as result of the oxidative uncoupling in the absence of thioNADP^+^ correspond with the initial velocities estimated for the ROS release (Fig. 3*E*) in the presence of saturating concentrations (1.0 mM) of NAD(P)H during the same reaction. The uncoupling observed for SthA aligns quite well with that reported for other different flavoproteins, such as acyl-coA dehydrogenase (31), phenylacetone monooxygenase, eugenol oxidase, and 5-hydroxymethyl furfural oxidase (19). All these enzymes exhibited uncoupling activity in addition to their canonical activity, releasing mainly H_2_O_2_ rather than superoxide. Evidently, the high redox potential of the O_2_/H_2_O_2_ couple of +0.27 V (32) provides a powerful driving force for reduced flavoproteins (with potentials between -0.40 and +0.13 V) (20, 33) to react with dioxygen, resulting in an electron leakage toward ROS formation. In the case of SthA, we even detected the formation of ROS taking place during the transhydrogenation from NADH to thioNADP^+^ (when oxygen was present in the reaction environment), leading us to determine the coupling efficiency as the amount of NADH hydrides transferred to the different products (thioNADPH, peroxide, superoxide anion). Therefore, we could assess that 98% of the electrons are destined for generating the reduced nicotinamide cofactor, while only 2% react with dioxygen.

When *udhA* gene of *E. coli* was characterized as encoding for a soluble transhydrogenase (34), it was highlighted the presence of a CXXXXT motif that distinguished STHs from the FDRs family, which instead displays a typical CXXXXC motif. The conservation of threonine in this motif of STHs has been further corroborated with the recent discovery of the first transhydrogenase with a SXXXXT domain in *Streptomyces avermitilis* (35). Nonetheless, no progress has been made to understand what biochemical role(s) lie behind the conservation of the CXXXXT motif among STHs. In this work, the investigation of SthA mutants that replace the cysteine (C45A) and restore the disulfide center of FDRs (T50C) was carried out to elucidate the roles of these amino acids (Fig. 4*A*). First, both single mutants showed somewhat lower FAD content after purification than WT SthA (Table 2), indicating that Cys45 and Thr50 promote tight flavin cofactor binding. Second, both C45A and T50C underwent a drastic (10-fold) decrease in the activity of mediating transhydrogenation between NADH and thioNADP^+^ (Fig. 4*C*), leading to the conclusion that the combination of Cys45 and Thr50 is strictly necessary for the optimal hydride transfer catalyzed by SthA. Third, the replacement of Cys45 and Thr50 differently affected the reductive half-reaction. On one side, the kinetic parameters of reduction of the protein-bound FAD by NADH (Table 3) revealed that C45A and T50C share a similar deterioration of the *K*_d_ value for NADH (around 3–4 times higher than WT), but they boosted the reduction rate for both mutant enzymes. The restoration of the cysteine in T50C led to a *k*_red_ value three and seven times higher than C45A and WT, respectively. On the other hand, the ability to act as a transhydrogenase decreased by one order of magnitude, which suggests that release of product or the rate of reoxidation of the FAD is slower in both mutants enzymes. Intriguingly, even though the kinetics for operating as a transhydrogenase was severely affected by each mutation, the ability to act as a NAD(P)H oxidase, generating ROS, was hardly affected. WT SthA reaches the maximum coupling efficiency toward the generation of thioNADPH (around 8.5 μmol min^−1^ mg^−1^), attenuating at the same time the aerobic ROS release to only 2%. When Cys45 or Thr50 are mutated, the thioNADPH formation remains prevalent (although never more than 10% of WT), but more electrons are diverted from NADH to the main uncoupling ROS product, H_2_O_2_.

To conclude, we have here provided new insights into the molecular functioning of SthA, a biocatalyst with appealing applications for metabolic engineering *in vivo* and *in vitro*. By cycling the redox status of the nicotinamide cofactors (14, 36), SthA allows to bypass the limitations imposed by the cofactor specificity that separates catabolic and anabolic enzymatic processes, which mostly require NAD(H) and NADP(H) respectively. Our characterization of SthA adds new information hitherto unexplored to optimally utilize the SthA-mediated NADPH-forming reaction, to take into account side activities such as uncoupling activity, and to minimize detrimental conditions to perform transhydrogenation, such as high concentrations of phosphate. Further research is needed to elucidate the role of SthA *in vivo* in light of these new properties emerged *in vitro*: for instance, the clarification of the relationship between SthA and oxidative stress in *E. coli*, since the 3 ′end of the *udhA* gene exhibits a 12-nucleotide overlap with the 3′ end of *oxyR* (Fig. S10) (34), a well-known H_2_O_2_-activated regulator of the cellular antioxidant response (37). This genetic arrangement (present in *E. coli* but not in *A. vinelandii* and *P. fluorescens*) might indicate some transcriptional regulation occurring under specific conditions between the two genes, as often indicated for overlapping genes (38, 39). Alternatively, considering the repression exerted by the bacterial response regulator ArcA (40) on the transcription of *udhA* during anaerobic respiration of *E. coli* (41, 42), it would be gainful to investigate the transhydrogenase role during the microaerophilic growth or the adaptive transition from aerobic to anaerobic environments and vice versa.

## Experimental procedures

### Chemicals

Unless specifically stated, all chemicals and enzymes were purchased at the highest purity grade available from Carl Roth GmbH & Co. KG or from Sigma-Aldrich (Merck, KGaA). PfuUltra Hotstart PCR Mastermix was purchased from Agilent Technologies, Inc. Primers were ordered from GATC/Eurofins Genomics.

### Protein overproduction and purification

*E. coli* MC 1061 (43) competent cells were transformed with the previously described expression vector pBXC3H_SthA (16), containing the *sthA* gene codifying the soluble transhydrogenase of *E. coli* K-12 (NCBI Gene ID: 948461). A preculture grown overnight at 37 °C was initially used to inoculate a 100 ml culture of sterile LB medium supplemented with 100 μg/ml ampicillin and then left to grow at 200 rpm at the same temperature. After reaching an optical density value between 0.5 and 0.7, the overexpression was induced by the addition of final 0.01% v/v L-arabinose and carried out for 20 h at 20 °C, shaking at 200 rpm. Then, the cells were centrifuged for 15 min, 6000*g*, 4 °C and the resulting yellow pellet was resuspended in lysis buffer (50 mM Tris at pH 7.5, 150 mM NaCl, 20 μM FAD) to be washed by centrifugation using the abovementioned parameters. After the pellet resuspension again in Lysis buffer, 1 mM PMSF (protease inhibitor), 100 μg/ml DNase, and 1 mM MgSO_4_ were added to the cells prior their disruption on ice with a VCX130 Vibra-Cell sonicator (Sonics & Materials, Inc) for 10 min (3 s on, 6 s off cycle, 70% amplitude). 1 mM potassium ethylenediaminetetraacetate at pH 7.0 was added to the lysate, which was then immediately centrifuged at 48254*g* for 30 min, 4 °C, to remove the insoluble material. The soluble fraction was left for 30 min at 4 °C under agitation to bind with 1 ml Nickel-sepharose resin (6 Fast Flow Cytiva)—previously washed with 20 column volumes (CV) Milli-Q and 20 CV SEC buffer (50 mM Tris at pH 7.5, 150 mM NaCl)—plus 10 mM imidazole at pH 7.5 to prevent aspecific binding. Then, the soluble fraction was let to flow through, washed with washing buffer (50 mM Tris at pH 7.5, 150 mM NaCl, 50 mM imidazole at pH 7.5), and consequently the protein bound to the resin material was eluted by adding consecutive volumes of elution buffer (50 mM Tris at pH 7.5, 150 mM NaCl, 500 mM imidazole at pH 7.5). The fractions containing the protein are clearly visible as they are yellow. The resulting protein from the affinity chromatography was first centrifuged for 5 min at 4 °C with 1 mM potassium ethylenediaminetetraacetate at pH 7.5 on a table-centrifuge (top speed) and consequently loaded on a Superdex 200 Increase 10/300 Gl column (GE Healthcare), attached to a FPLC-system (Bio-Rad Laboratories, Inc) allowing the simultaneous detection of the wavelengths at 280 and 450 nm. The protein elution on SEC was carried out with SEC buffer, and the fractions correspondent to the peak were pooled and concentrated by using centrifugal devices (Amicon Ultra 0.5 ml, Merck Millipore Ltd) with a 30 kDa cut-off. Once obtained a concentration around 2 to 4 mg ml, glycerol was added reaching final 10% (v/v) of the protein sample. After carefully mixing, the protein concentration was reassessed by measuring on a spectrophotometer Cary 100 Bio (Varian) the absorbance at 450 nm and applying this value to the Lambert-Beer equation with the calculated extinction coefficient (ε_450_ = 12.1 mM^−1^ cm^−1^) for SthA. Usually, the whole absorbance spectrum from 200 to 700 nm was recorded at room temperature to verify the sample quality. Last, the SthA aliquots were flash-frozen in liquid nitrogen and stored at −80 °C.

### Dynamic light scattering

Following protein purification, 8 μl of SthA (>1 mg/ml) were loaded on a cyclic olefin copolymers microcuvette and used for DLS measurements on a DynaPro Nanostar (Wyatt technology). The analysis on three samples from independent purification batches (*n* = 3) was carried out to determine the molar mass and the polydispersity of the purified enzyme (Fig. 1*D*). For each sample, we acquired a final DLS distribution coming from the average of 10 acquisitions every 20 s at the constant temperature of 20 °C.

### Site-directed mutagenesis

The site-directed mutagenesis was performed by using the plasmid pBXC3H_SthA as a template, containing the gene encoding for the WT protein. The QuikChange method was applied according to the manufacturer instructions (Agilent Technologies, Inc). Primers were designed by using the Agilent QuikChange primer design tool (http://www.genomics.agilent.com/primerDesignProgram.jsp). The sequences of the primers are listed in Table S2. The final PCR reaction mixture consisted of final 25 μl and contained 20 to 30 ng template, 0.2 μM forward and reverse primer, and 1× PfuUltra II Hotstart PCR Master Mix (already incorporating PCR reaction buffer, magnesium, and dNTPs). The mixture was subjected to the following PCR conditions: 95 °C for 3 min, 30 cycles of 95 °C for 30 s, 60 °C for 30 s and 72 °C for 3 min, and a final extension at 72 °C for 5 min. The PCR products were digested by DpnI at 37 °C for 30 min to remove the parental templates, after which the reaction mixtures were used to transform chemically competent *E. coli* MC 1061. The mutations were verified by commercial sequencing at GATC/Eurofins Genomics. The resulting plasmids pBXC3H_SthAT50C and pBXC3H_SthAC45A were then employed to transform chemocompetent *E. coli* MC 1061 cells in order to carry out the protein overproduction and purification as described for the WT enzyme.

### Transhydrogenation steady-state kinetics and activity assays

The catalytic transhydrogenation mediated by SthA was measured at the single wavelength of 400 nm as thioNADPH formation (ε_400 nm_ = 11.7 mM^−1^ cm^−1^ (44)), according to the Lambert-Beer law, in a 96-well plate reader (SPARK 10M, Tecan). Setting the temperature control at 30 °C, the steady-state kinetic experiments were carried out in activity buffer (50 mM Tris, pH 7.5) using three independent purification batches of SthA, freshly diluted prior of the measurement. The addition of 10 μl of 0.2 μM SthA (final 10 nM) to 190 μl mixture containing activity buffer, NADH, and thioNADP^+^ was starting the reaction, for a final volume of 200 μl. To determine the kinetic parameters of NADH, we varied the concentration of the reduced cofactor from 0.1 to 18.0 mM, while thioNADP^+^ was fixed at 500 μM. For the determination of the kinetic values of thioNADP^+^, 10 to 750 μM was the tested concentration range of the thio-nicotinamide, whereas NADH was kept constant at 10.0 mM. The resulting initial velocities were fit according to the “Substrate-Inhibition” kinetic models implemented in the software GraphPad Prism 5.0 (GraphPad Software Inc) according to the (Equation 2):

(2) $$v=\frac{V_{\text{MAX}}\text{∗}\left[\text{S}\right]}{K_{\text{M}}+\left[\text{S}\right]\text{∗}\left(1+\frac{\left[\text{S}\right]}{K_{\text{I}}}\right)}$$

in which *v* represents the initial velocity, [S] is the substrate concentration, and $K_{\text{I}}$ stands for the inhibitory constant.

The pH optimum experiments (Fig. 2*A*) were performed using freshly prepared 1 M buffers, then diluted to 0.1 M with Milli-Q. Tested buffers were acetate, KPi, Tris/HCl, and carbonate/bicarbonate. The reaction wells were filled with 150 μl buffer of choice, 20 μl 1.0 mM thioNADP^+^ and 20 μl of freshly diluted 200 nM SthA, and incubated for 2 min at 30 °C. The addition of 10 μl of 10.0 mM NADH was starting the reaction (final concentrations: 100 mM buffer, 20 nM SthA, 0.1 mM thioNADP^+^, 0.5 mM NADH), which was monitored spectrophotometrically at 400 nm. The condition corresponding to the higher initial velocity—100 mM Tris at pH 8.0—was set arbitrarily as 100% activity. The dataset comes from four independent measurements (*n* = 4) using different protein purification batches for single technical replicates, while the error bars display the s.e.m.

The phosphate effect on thioNADPH formation (Fig. 2*B*) was assessed in the same way of the pH optimum setup, fixing the initial velocity of SthA—corresponding to the first minute of reaction—in 50 mM Tris at pH 7.5 (Activity buffer) as 100% relative activity, and comparing the change in velocity in the presence of different amounts of KPi or sodium phosphate at pH 7.5. In this case, the cofactor concentrations were changed in 0.15 mM thioNADP^+^ and 1.0 mM NADH, while the measurements were done in biological triplicate, each of them with a single technical replicate (*n* = 3, error bars represent the s.e.m.). The ionic strength experiments (Fig. S4) were done in the same way, by employing increasing quantities of NaCl or KCl. The effect of adenine nucleotides (Fig. 2*D* and Table S1) was assessed in a similar manner, as the assay was carried out at 30 °C in a reaction mixture including activity buffer, 5.0 mM of one between AMP/ADP/ATP (freshly prepared and solubilized in the same buffer), 0.15 mM thioNADP^+^ and 1.0 mM NADH, triggered by the addition of 20 nM SthA variant of interest (alternatively WT, T50C, or C45A).

### Uncoupling oxidase activity and ROS formation measurements

In the case of the determination of the kinetic parameters for the oxidase activity (Table 1), the experimental setup was similar to the steady-state kinetics for the transhydrogenation. The oxidation of NAD(P)H was monitored at 340 nm (ε_340 nm_ = 6.22 mM^-1^ cm^−1^) and keeping the temperature constant at 30 °C while testing different concentrations of the reduced cofactor from to 10 to 400 μM. The experiments were started by injecting 10 μl of 12.0 μM SthA (final 600 nM) to 190 μl mixture containing activity buffer and NADH or NADPH at the chosen concentration. The kinetic plots shown in Fig. S6 represent the average of three independent repetitions (different purification batches).

The superoxide anion detection (Figs. 3, *D*, *E*, 4, *C* and *D*) was performed spectrophotometrically by monitoring the reduction of cytochrome *c* (Fe^3+^) from equine heart (Sigma-Aldrich, C7752) into its ferrous state (cytochrome *c* Fe^2+^, ε_550 nm_ = 21.0 mM^−1^ cm^−1^ (45)) at the wavelength of 550 nm. Briefly, the oxidase or transhydrogenation activities were followed by including final 20 μM cytochrome *c* in the reaction well of the microplate and measuring the increase in absorbance over time, which was then converted into formed superoxide by Lambert-Beer's law. Each condition had a negative control without the addition of 600 nM SthA, in order to blank any aspecific electron transfer from the excess of reduced cofactors. In particular, for the uncoupling activity, we used 1.0 mM NADH or NADPH, while for the transhydrogenation, we included 1.0 mM NADH and 0.15 mM thioNADP^+^. In both the cases, the reactions were carried out in activity buffer for 10 min, at 30 °C, in a 200 μl reaction volume under continuous shaking (270 rpm) and started by adding final 600 nm SthA (WT, C45A, or T50C).

The generation of H_2_O_2_ was quantified using the xylenol orange method (46). A xylenol stock solution (125 μM xylenol orange, 100 mM D-sorbitol) was prepared and freshly mixed in the ratio 99:1 with the ferrous solution (25 mM (NH_4_)_2_Fe(SO_4_)_2_, 2.5 M H_2_SO_4_), obtaining the final xylenol assay solution (125 μM xylenol orange, 100 mM D-sorbitol, 250 μM (NH_4_)_2_Fe(SO_4_)_2_, 25 mM H_2_SO_4_). For different time points of both the uncoupling or transhydrogenase reactions (carried out as described for the superoxide assays), 20 μl aliquots of the reactions were added and mixed to 200 μl of the xylenol assay solution and let to incubate for 15 min at 30 °C in the dark. Also in this case, each reaction had a correspondent control devoid of any SthA addition in order to blank any possible aspecific reaction for each time point (1, 3, 5, 10 min). Immediately upon incubation, we measured the samples absorbance at 595 nm and estimated the amount of H_2_O_2_ removing the blank contribution to the absorbance values. The quantification of formed peroxide (Figs. 3*D* and 4*E*) was carried out by interpolation with calibration curves of H_2_O_2_ in the presence of the xylenol assay solution, treated in the same manner described for the enzymatic reactions. The calculation of the initial velocity values (Figs. 3*E* and 4*C*), corresponding to the first minute of reaction, were estimated by Lambert-Beer law utilizing the extinction coefficient corresponding to 15.0 mM^-1^ cm^−1^ for xylenol in 25 mM H_2_SO_4_ (47).

### Stopped-flow measurements

All experiments were performed by a SX20 stopped-flow spectrophotometer equipped with a photodiode array detector or in the single channel photomultiplier (PMT) mode (Applied Photophysics). To investigate the reoxidation of the reduced flavoprotein (Fig. 3*B*), 7.5 or 10.0 μM SthA WT were mixed with 50 μM NADH in aerobical or anaerobical conditions. For the anaerobic reaction, the machine was first balanced and filled with freshly degassed buffer 50 mM Tris–HCl buffer, pH 7.5, 150 mM NaCl, and 5% glycerol. Then, all the components containing 5 mM glucose were gently flushed with nitrogen for 10 min and consequently mixed with 0.3 μM glucose oxidase to remove the dissolved oxygen. After mixing, the reaction was followed by monitoring the absorbance change at full wavelength (photodiode array detector) or a single wavelength (PMT). To determine the rate of flavin reduction (Table 3, Figs. *D* and S9), the single channel PMT mode was used and the wavelength was set at 450 nm. Four to eight micromolar enzyme (WT, T50C, or C45A) were mixed with varying concentrations of NADH (5–250 μM) at 25 °C. The absorbance value at 450 nm was monitored over time and then converted into observed slopes (*k*_obs_) by using Pro-data viewer (Applied Photophysics) assuming a single exponential model. All experiments were performed in biological triplicate. The *k*_obs_ values were plotted against the NADH concentration and fitted with the Michaelis−Menten equation in Graphpad.

### Phylogenetic analyses

Homology searches were performed using *E. coli* sequence (GenBank MBB9749438) as query in Blastp. Searches were performed restricting the taxonomy to the three domains of life separately. When searching in Archaea, no STHs were found, but instead dihydrolipoyl dehydrogenases sequences were retrieved. These were collected and included in the dataset as outgroup. All hits reviewed in SwissProt and those belonging to experimentally characterized STHs were selected for further analysis. A multiple sequence alignment (MSA) was constructed in MAFFT7 (48). Single sequence extensions and insertions were trimmed and the resulting MSA (60 seqs and 469 sites) was used to infer the phylogeny (Fig. S7). The tree was inferred by Maximum Likelihood inference method in RAxML v8.2.10 (500 rapid bootstraps) and transfer bootstrap expectation was performed in Booster (49). An MSA containing only STHs was also built and used to obtain a sequence logo (Fig. 4*A*) with the online tool WebLogo3 (50) to evidence the presence of the canonical motifs in the protein family.

### Structural analyses

The AlphaFold-predicted structure of SthA from *E. coli* K-12 (identifier AF-B7M718-F1) was extracted from the AlphaFold Protein Structure database (51). The FAD and NAD^+^ cofactors were docked using PyMOL (52), by the superimposition of the crystallographic holo-structure of the lipoamide dehydrogenase from *Thermus thermophilus* HB8, (PDB entry:2EQ7) with the predicted apo-structure of SthA.

## Data availability

All relevant data are available in the main article or in the correspondent supporting information. The predicted AlphaFold structure of SthA (UniProt: P27306) is available on the AlphaFold Protein Structure database with the identifier AF-B7M718-F1.

## Supporting information

This article contains supporting information. Figures S1–S10 and Supplemental Tables 1 and 2 are available in the Supporting Information document. The supplemental figures include loss of FAD during SthA purification on SEC, molecular weight of purified SthA after SEC, visualization of purified SthA by Transmission Electron Microscopy, the effect of increasing ionic strength on SthA transhydrogenation, changes in the absorbance spectrum of SthA upon NADH addition in the absence of an oxidized nicotinamide adenine dinucleotide, kinetics curves of the oxidase activity of SthA with NADH and NADPH, phylogeny of representative STHs, SEC profiles of the C45A and T50C mutants, absorbance spectrum of the SthA constructs upon NADH addition in the absence of oxygen or any other oxidized nicotinamide cofactor, and the overlap between the *udhA* and *oxyR* genes in *E. coli* K-12 genome. The supplemental tables report the percentual activity of WT, T50C, and C45A is activated by ATP, ADP, and AMP, the primers for the mutagenesis of the CXXXXT motif. The supplementary experimental procedures include reporting the negative staining and electron microscopy of SthA.

## Conflict of interest

The authors declare that there are no conflicts of interest with the contents of this article.

## References

[1] Hoek J.B., Rydstrom J. (1988). Physiological roles of nicotinamide nucleotide transhydrogenase. Biochem. J..

[2] Blank L.M., Ebert B.E., Buehler K., Bühler B. (2010). Redox biocatalysis and metabolism: molecular mechanisms and metabolic network analysis. Antioxid. Redox Signal..

[3] Jackson J.B. (2003). Proton translocation by transhydrogenase. FEBS Lett..

[4] Argyrou A., Blanchard J.S. (2004). Flavoprotein disulfide reductases: advances in chemistry and function. Prog. Nucl. Acid Res. Mol. Biol..

[5] Kleiger G., Eisenberg D. (2002). GXXXG and GXXXA motifs stabilize FAD and NAD(P)-binding rossmann folds through Cα-H···O hydrogen bonds and van der waals interactions. J. Mol. Biol..

[6] Sauer U., Canonaco F., Heri S., Perrenoud A., Fischer E. (2004). The soluble and membrane-bound transhydrogenases UdhA and PntAB have divergent functions in NADPH metabolism of *Escherichia coli*. J. Biol. Chem..

[7] Zhao H., Wang P., Huang E., Ge Y., Zhu G. (2008). Physiologic roles of soluble pyridine nucleotide transhydrogenase in *Escherichia coli* as determined by homologous recombination. Ann. Microbiol..

[8] Sánchez A.M., Andrews J., Hussein I., Bennett G.N., San K.Y. (2006). Effect of overexpression of a soluble pyridine nucleotide transhydrogenase (UdhA) on the production of poly(3-hydroxybutyrate) in *Escherichia coli*. Biotechnol. Prog..

[9] Wang Z., Gao C., Wang Q., Liang Q., Qi Q. (2012). Production of pyruvate in Saccharomyces cerevisiae through adaptive evolution and rational cofactor metabolic engineering. Biochem. Eng. J..

[10] Jan J., Martinez I., Wang Y., Bennett G.N., San K.Y. (2013). Metabolic engineering and transhydrogenase effects on NADPH availability in *Escherichia coli*. Biotechnol. Prog..

[11] Xu W., Yao J., Liu L., Ma X., Li W., Sun X. (2019). Improving squalene production by enhancing the NADPH/NADP+ ratio, modifying the isoprenoid-feeding module and blocking the menaquinone pathway in *Escherichia coli*. Biotechnol. Biofuels..

[12] Shi T., Han P., You C., Zhang Y.H.P.J. (2018). An *in vitro* synthetic biology platform for emerging industrial biomanufacturing: bottom-up pathway design. Synth. Syst. Biotechnol..

[13] Bowie J.U., Sherkhanov S., Korman T.P., Valliere M.A., Opgenorth P.H., Liu H. (2020). Synthetic biochemistry: the bio-inspired cell-free approach to commodity chemical production. Trends Biotechnol..

[14] Boonstra B., Rathbone D.A., French C.E., Walker E.H., Bruce N.C. (2000). Cofactor regeneration by a soluble pyridine nucleotide transhydrogenase for biological production of hydromorphone. Appl. Environ. Microbiol..

[15] Mouri T., Shimizu T., Kamiya N., Goto M., Ichinose H. (2009). Design of a cytochrome P450BM3 reaction system linked by two-step cofactor regeneration catalyzed by a soluble transhydrogenase and glycerol dehydrogenase. Biotechnol. Prog..

[16] Partipilo M., Ewins E.J., Frallicciardi J., Robinson T., Poolman B., Slotboom D.J. (2021). Minimal pathway for the regeneration of redox cofactors. JACS Au.

[17] Cao Z., Song P., Xu Q., Su R., Zhu G. (2011). Overexpression and biochemical characterization of soluble pyridine nucleotide transhydrogenase from *Escherichia coli*. FEMS Microbiol. Lett..

[18] Chaiyen P., Fraaije M.W., Mattevi A. (2012). The enigmatic reaction of flavins with oxygen. Trends Biochem. Sci..

[19] Gran-Scheuch A., Parra L., Fraaije M.W. (2021). Systematic assessment of uncoupling in flavoprotein oxidases and monooxygenases. ACS Sustain. Chem. Eng..

[20] Mattevi A. (2006). To be or not to be an oxidase: challenging the oxygen reactivity of flavoenzymes. Trends Biochem. Sci..

[21] Aliverti A., Curti B., Vanoni M.A. (1999). Identifying and quantitating FAD and FMN in simple and in iron-sulfur-containing flavoproteins. Met. Mol. Biol..

[22] RydströM J., Hoek J.B., Ernster L. (1976). 2 nicotinamide nucleotide transhydrogenases. Enzyme.

[23] Messner K.R., Imlay J.A. (2002). Mechanism of superoxide and hydrogen peroxide formation by fumarate reductase, succinate dehydrogenase, and aspartate oxidase. J. Biol. Chem..

[24] van Berkel W.J., Benen J.A., Eppink M.H., Fraaije M.W. (1999). Flavoprotein kinetics. Flavoprotein Protoc..

[25] Valentino H., Sobrado P. (2019). Performing anaerobic stopped-flow spectrophotometry inside of an anaerobic chamber. Met. Enzymol..

[26] Butler J., Jayson G.G., Swallow A.J. (1975). The reaction between the superoxide anion radical and cytochrome c. Biochim. Biophys. Acta.

[27] Kaplan N.O., Colowick S.P., Neufeld E.F. (1952). Pyridine nucleotide transhydrogenase: II. Direct evidence for and mechanism of the transhydrogenase reaction. J. Biol. Chem..

[28] Chung A.E. (1970). Pyridine nucleotide transhydrogenase from *Azotobacter vinelandii*. J. Bacteriol..

[29] Boonstra B., Bjo L., French C.E., Wainwright I., Bruce N.C. (2000). Cloning of the sth gene from *Azotobacter vinelandii* and construction of chimeric soluble pyridine nucleotide transhydrogenases. FEMS Microbiol. Lett..

[30] van den Broek H.W.J., van Breemen J.F.L., van Bruggen E.F.J., Veeger C. (1971). Pyridine-nucleotide transhydrogenase: 2. Electron-Microscopic studies on the transhydrogenase from *Azotobacter vinelandii*. Eur. J. Biochem..

[31] Wang R., Thorpe C. (1991). Reactivity of medium-chain acyl-CoA dehydrogenase toward molecular oxygen. Biochemistry.

[32] Massey V. (1994). Activation of molecular oxygen by flavins and flavoproteins. J. Biol. Chem..

[33] Heuts D.P.H.M., Scrutton N.S., McIntire W.S., Fraaije M.W. (2009). What’s in a covalent bond?: on the role and formation of covalently bound flavin cofactors. FEBS J..

[34] Boonstra B., French C.E., Wainwright I.A.N., Bruce N.C. (1999). The udhA gene of *Escherichia coli* encodes a soluble pyridine nucleotide transhydrogenase. J. Bacteriol..

[35] Cao Z., Liu J., Meng R., Wang P., Zhu G. (2022). Identification and characterization of a novel soluble pyridine nucleotide transhydrogenase from *Streptomyces avermitilis*. Curr. Microbiol..

[36] Liu J., Li H., Zhao G., Caiyin Q., Qiao J. (2018). Redox cofactor engineering in industrial microorganisms: strategies, recent applications and future directions. J. Ind. Microbiol. Biotechnol..

[37] Storz G., Tartaglia L.A. (1992). OxyR: a regulator of antioxidant genes. J. Nutr..

[38] Rogozin I.B., Makarova K.S., Murvai J., Czabarka E., Wolf Y.I., Tatusov R.L. (2002). Connected gene neighborhoods in prokaryotic genomes. Nucl. Acids Res..

[39] Huvet M., Stumpf M.P.H. (2014). Overlapping genes: a window on gene evolvability. BMC Genomics.

[40] Gunsalus R.P., Park S.J. (1994). Aerobic-anaerobic gene regulation in Escherichia coli: control by the ArcAB and fnr regulons. Res. Microbiol..

[41] Haverkorn van Rijsewijk B.R.B., Kochanowski K., Heinemann M., Sauer U. (2016). Distinct transcriptional regulation of the two *Escherichia coli* transhydrogenases PntAB and UdhA. Microbiol..

[42] Federowicz S., Kim D., Ebrahim A., Lerman J., Nagarajan H., Cho B.K. (2014). Determining the control circuitry of redox metabolism at the genome-scale. PLoS Genet..

[43] Casadaban M.J., Cohen S.N. (1980). Analysis of gene control signals by DNA fusion and cloning in *Escherichia coli*. J. Mol. Biol..

[44] Anderson B.M., Anderson C.D., Lee J.K., Stein A.M. (1963). The Thionicotinamide analogs of DPN and TPN. II. Enzyme studies. Biochemistry.

[45] Catucci G., Gao C., Rampolla G., Gilardi G., Sadeghi S.J. (2019). Uncoupled human flavin-containing monooxygenase 3 releases superoxide radical in addition to hydrogen peroxide. Free Radic. Biol. Med..

[46] Robinson R., Sobrado P. (2011). Substrate binding modulates the activity of *Mycobacterium smegmatis* G, a flavin-dependent monooxygenase involved in the biosynthesis of hydroxamate-containing siderophores. Biochemistry.

[47] Kitatsuji C., Izumi K., Nambu S., Kurogochi M., Uchida T., Nishimura S.I. (2016). Protein oxidation mediated by heme-induced active site conversion specific for heme-regulated transcription factor, iron response regulator. Sci. Rep..

[48] Katoh K., Rozewicki J., Yamada K.D. (2019). MAFFT online service: multiple sequence alignment, interactive sequence choice and visualization. Brief. Bioinform..

[49] Ehman E.C., Johnson G.B., Villanueva-meyer J.E., Cha S., Leynes A.P., Eric P. (2018). Renewing Felsenstein’s phylogenetic bootstrap in the era of big data. Nature.

[50] Crooks G.E., Hon G., Chandonia J.M., Brenner S.E. (2004). WebLogo: a sequence logo generator. Genome Res..

[51] Jumper J., Evans R., Pritzel A., Green T., Figurnov M., Ronneberger O. (2021). Highly accurate protein structure prediction with AlphaFold. Nature.

[52] WL D. (2002). The PyMOL Molecular Graphics System. Wiley Inter. Rev..

[53] Reynafarje B., Costa L.E., Lehninger A.L. (1985). O2 solubility in aqueous media determined by a kinetic method. Anal. Biochem..

