# Supplementary material for: A conserved sequence motif in the Escherichia coli soluble FAD-containing pyridine nucleotide transhydrogenase is important for reaction efficiency
Source: J Biol Chem. 2022 Aug 4;298(9):102304. doi: 10.1016/j.jbc.2022.102304 (PMC9460512; doi:10.1016/j.jbc.2022.102304)
Supplement: Supplementary information [file mmc1.docx]

Supplementary information for:

**Unraveling the unexplored biochemical properties of the soluble FAD-containing pyridine nucleotide transhydrogenase of *Escherichia coli***

**Michele Partipilo^1^, Guang Yang^2^, Maria Laura Mascotti^2,3^, Hein J. Wijma^2^, Dirk Jan Slotboom^1^, and Marco W. Fraaije^2^**

^1 Membrane Enzymology Group, Groningen Institute of Biomolecular Sciences & Biotechnology, University of Groningen, Nijenborgh 4, 9747 AG Groningen, The Netherlands.^

^2 Molecular Enzymology Group, Groningen Institute of Biomolecular Sciences & Biotechnology, University of Groningen, Nijenborgh 4, 9747 AG Groningen, The Netherlands.^

^3 IMIBIO-SL CONICET, Facultad de Química Bioquímica y Farmacia, Universidad Nacional de San Luis, Ejercito de los Andes 950, D5700HHW, San Luis, Argentina.^

This file contains:

- **Supplementary Figures S1-S10:**

Loss of FAD during SthA purification on SEC (**Fig. S1**), molecular weight of purified SthA after SEC (**Fig. S2**), visualization of purified SthA by Transmission Electron Microscopy (**Fig. S3**), the effect of increasing ionic strength on SthA transhydrogenation (**Fig. S4**), changes in the absorbance spectrum of SthA upon NADH addition in the absence of an oxidized nicotinamide adenine dinucleotide (**Fig. S5**), kinetics curves of the oxidase activity of SthA with NADH and NADPH (**Fig. S6**), phylogeny of representative STHs (**Fig. S7**), SEC profiles of the C45A and T50C mutants (**Fig. S8**), absorbance spectrum of the SthA constructs upon NADH addition in the absence of oxygen or any other oxidized nicotinamide cofactor (**Fig. S9**), the overlap between the *udhA* and *oxyR* genes in *E. coli* K-12 genome (**Fig. S10**).

- **Supplementary Tables 1-2:**

The percentual activity of WT, T50C and C45A is activated by ATP, ADP and AMP (**Table 1**), the primers for the mutagenesis of the CXXXXT motif (**Table 2**).

- **Supplementary Experimental procedures:**

Negative staining and electron microscopy of SthA.

**Supplementary Figures**


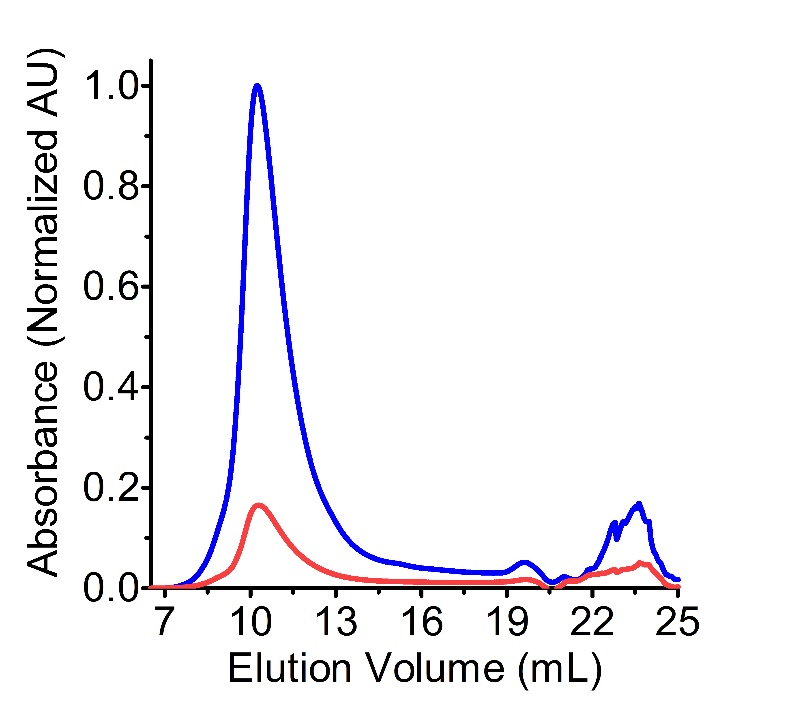


**Supplemental Figure 1** Loss of FAD during SthA purification on SEC. The protein elution on a Superdex 200 Increase 10/300 is monitored at 280 nm (blue line) and 450 nm (red line). The broad peak between 21 and 25 mL corresponds to FAD (MW 782.53 Da), beyond the limit of the efficient separation ensured by the column material.


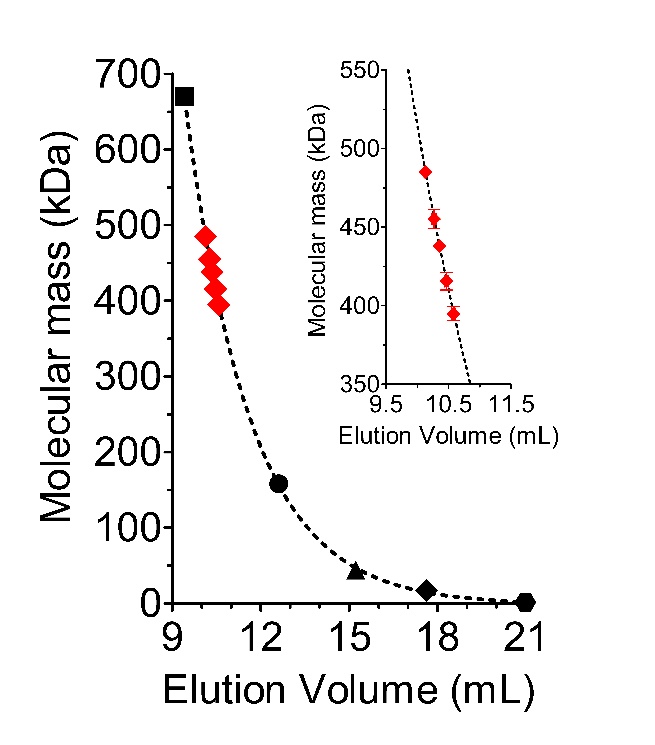


**Supplemental Figure 2** Molecular weight of purified SthA after SEC. 5 independent overproduction batches of SthA (*n* = 5, error bands for each point correspond to the standard deviation between the maximum peak obtained at 280 nm and the maximum absorbance at 450 nm) were purified according to the described protocol in the specific “Materials and Methods” section of the main manuscript, and loaded on a Superdex 200 Increase 10/300 size exclusion column. The calibration line for the molecular mass according to the elution volume was carried out using the following gel filtration standards: ■ Thyroglobulin 670 kDa, ● Gamma globulin 158 kDa, ▲ Ovalbumin 44 kDa, ♦ Myoglobin 17 kDa, ⬣ Vitamin B12 1 kDa.


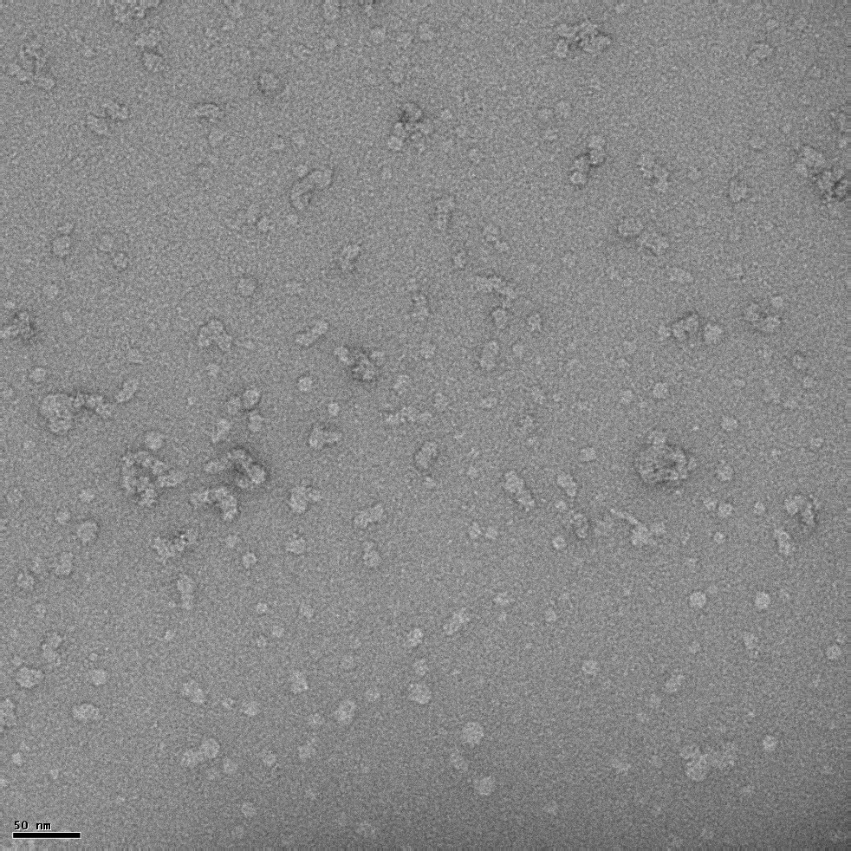


**Supplemental Figure 3** Visualization of purified SthA by Transmission Electron Microscopy (TEM). The freshly purified protein was diluted to 0.01 mg mL^-1^ and then stained with 2% uranyl acetate on a carbon-coated grid to be visualized on a 120 kV electron microscope. The scale bar corresponds to 50 nm.


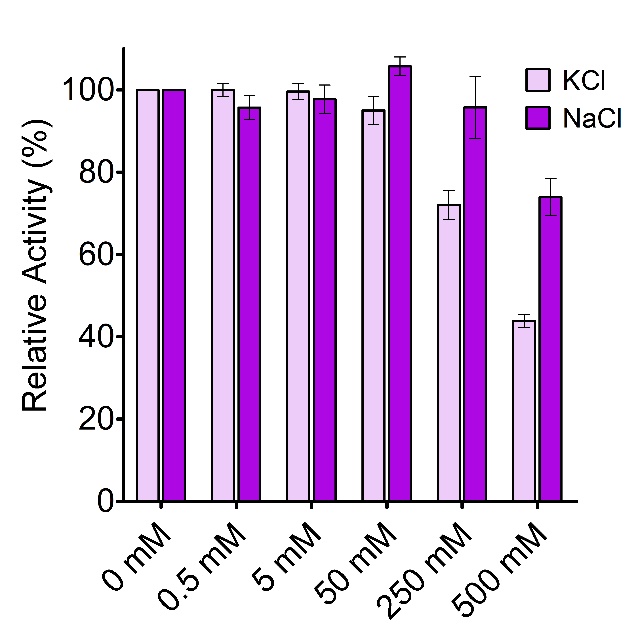


**Supplemental Figure 4** The effect of increasing ionic strength on SthA transhydrogenation. Increasing the amount of NaCl (purple bars) or KCl (pink bars), we did not observed a significant change in the transhydrogenation rate up to 50 mM salt. Only very high ionic strength affected the protein activity, reaching 70% in the presence of 500 mM NaCl or 40% with 500 mM KCl. 100% activity was fixed as the transhydrogenation rate reached at 30 °C in the reaction mixture devoid of any salt and composed of 50 mM Tris at pH 7.5 (Activity buffer), 20 nM SthA, 0.15 mM thioNADP^+^ and 1.0 mM NADH (*n* = 3, error bars display the s.e.m.).


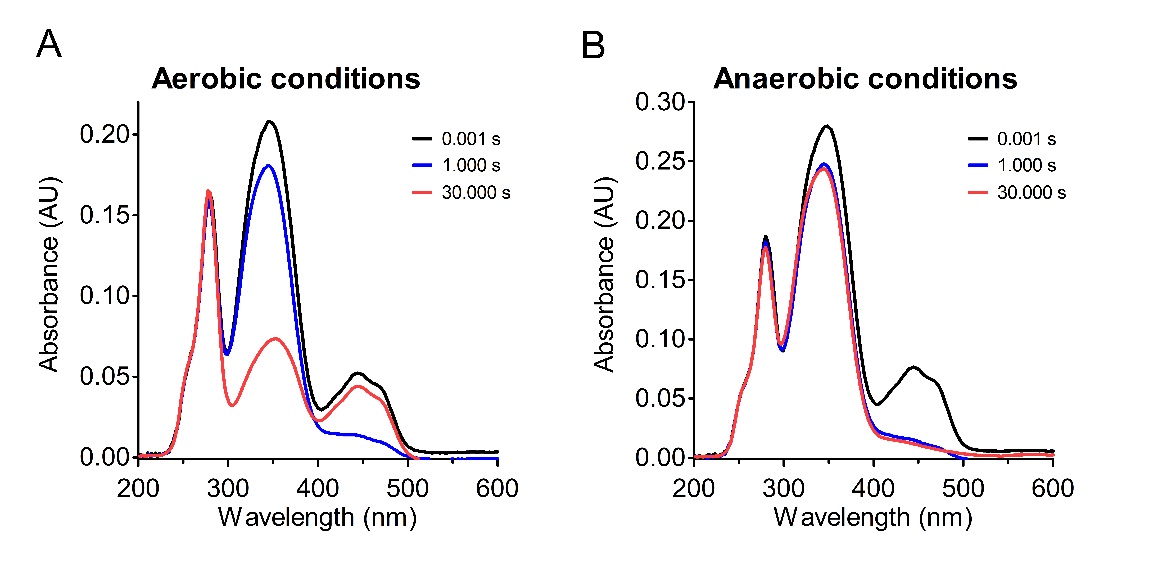


**Supplemental Figure 5** Changes in the absorbance spectrum of SthA upon NADH addition in the absence of an oxidized nicotinamide adenine dinucleotide. In the presence of oxygen (**A**), mixing 50 µM NADH with 7.5 µM SthA leads to the oxidation of the nicotinamide cofactor over time (decrease of the absorbance peak at 340 nm). Such an oxidative event is made possible by the transient reduction of the prosthetic FAD, visible as decrease of the peak at 450 nm (after 1 second of reaction, blue line), followed by the flavin reoxidation as recovery of the absorbance value at 450 nm within 30 seconds (red line). When the reaction takes place in anaerobiosis (**B**), the same amount of NADH is not fully oxidized by 10.0 µM SthA. Due to the lack of O_2_ as final electron acceptor, the reducing equivalents are retained by the embedded-FAD of the flavoenzyme (lack of the peak at 450 nm after 30 seconds of reaction, red line), which cannot be reoxidized to ensure further oxidative cycles of the uncoupling activity.


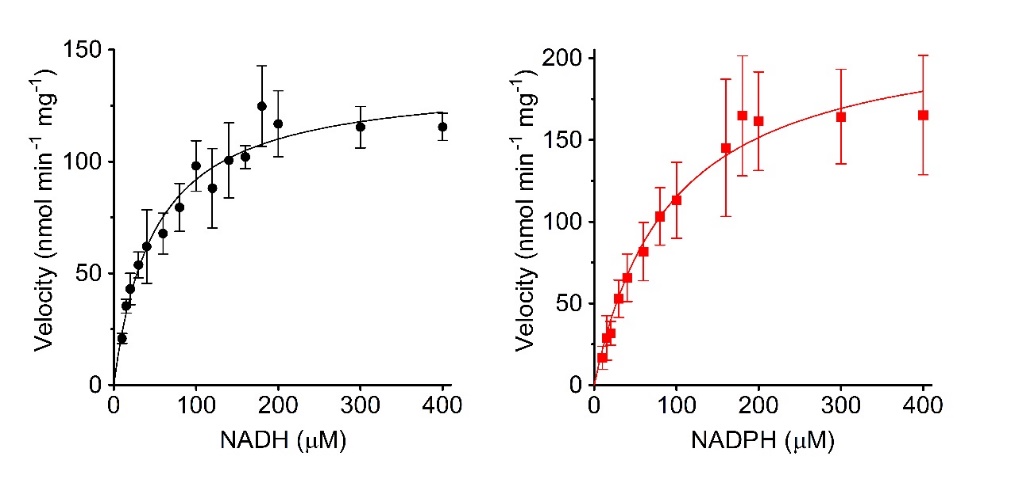


**Supplemental Figure 6** Kinetics curves of the oxidase activity of SthA with NADH and NADPH. The data comes from 3 independent purification batches (*n* = 3, errors bars display the s.e.m.) and they are plotted according to the Michaelis-Menten model. Assuming the fixed amount of O_2_ in aqueous solutions at 0.2 mM, we estimated for NADH (on the left, in black) a *K*_M_ of 49.09 ± 10.37 µM, a *V*_MAX_ of 137.1 ± 8.8 nmol min^-1^ mg^-1^, and a *k*_CAT_ of 0.12 ± 0.01 s^-1^. For NADPH (on the right, in red), we calculated a *K*_M_ of 91.76 ± 30.46 μM, the *V*_MAX_ at 221.0 ± 28.3 nmol min^-1^ mg^-1^, and a *k*_CAT_ of 0.20 ± 0.03 s^-1^. The kinetic experiments were carried out at 30 °C in 50 mM Tris at pH 7.5 employing 600 nM SthA. The rates were obtained by Lambert-Beer’s law using the extinction coefficient at 340 nm of NAD(P)H (ε_NAD(P)H_ = 6.22 mM^-1^ cm^-1^).

**
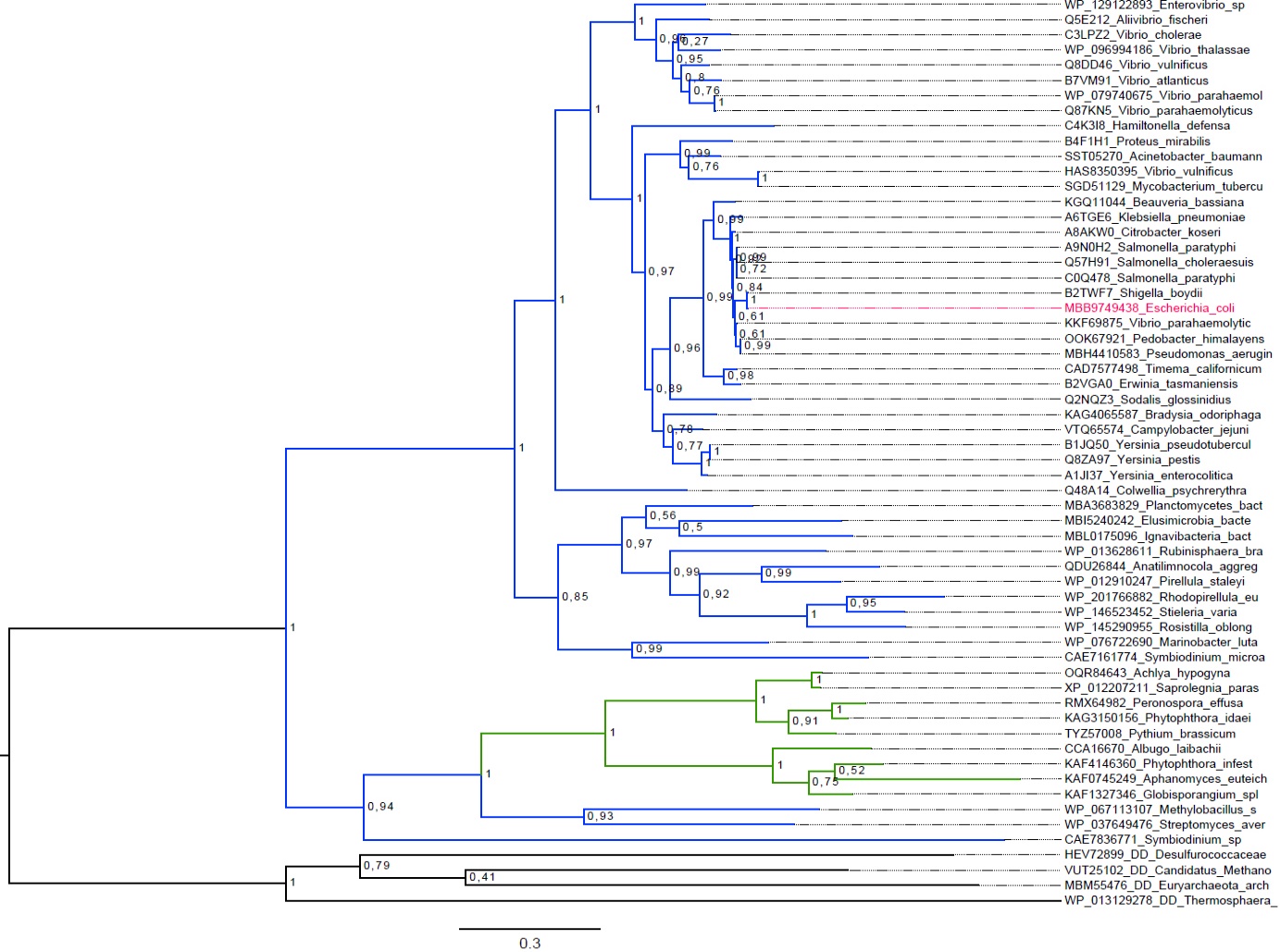
**

**Supplemental Figure 7** Phylogeny of representative STHs. The tree was constructed in RAxML v8.2.10 with 500 rapid bootstrap (BS). BS were subjected to transfer (TBE) and TBE values are shown at the nodes. To date, STHs were thought to be encoded only in some bacteria, while we identified a few STHs from the Oomycota phylum. The clade of bacterial STHs is shown with blue branches and the newly discovered clade from Eukarya in green. The sequence investigated here is highlighted in pink letters. The tree was rooted at the clade formed by the archaeal dihydrolipoyl dehydrogenases (DDs are represented as black branches).


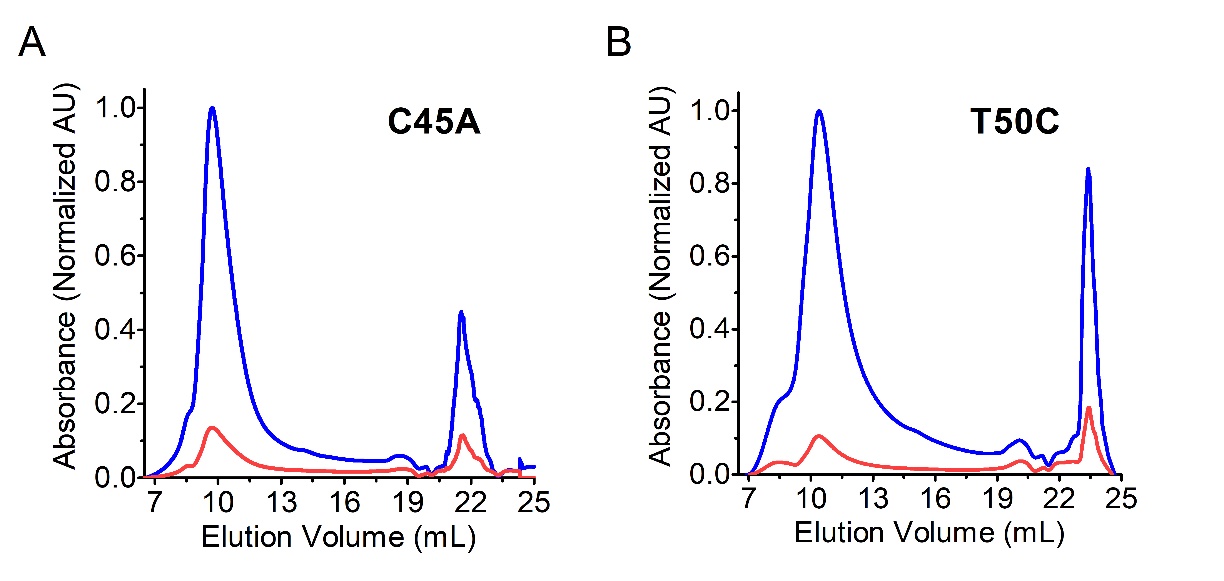


**Supplemental Figure 8** SEC profiles of the C45A (**A**) and T50C (**B**) mutants. Both the variants elute on a Superdex 200 Increase 10/300 in the correspondence of the same elution volume of the WT protein (Fig. S1). Nonetheless, the loss of FAD (peak between 21 and 25 mL, for a molecular weight of 782.53 Da) over the purification procedure is 2-fold and 4-fold higher respectively in C45A and T50C. The protein elution is monitored at 280 nm (blue line) and 450 nm (red line).

**
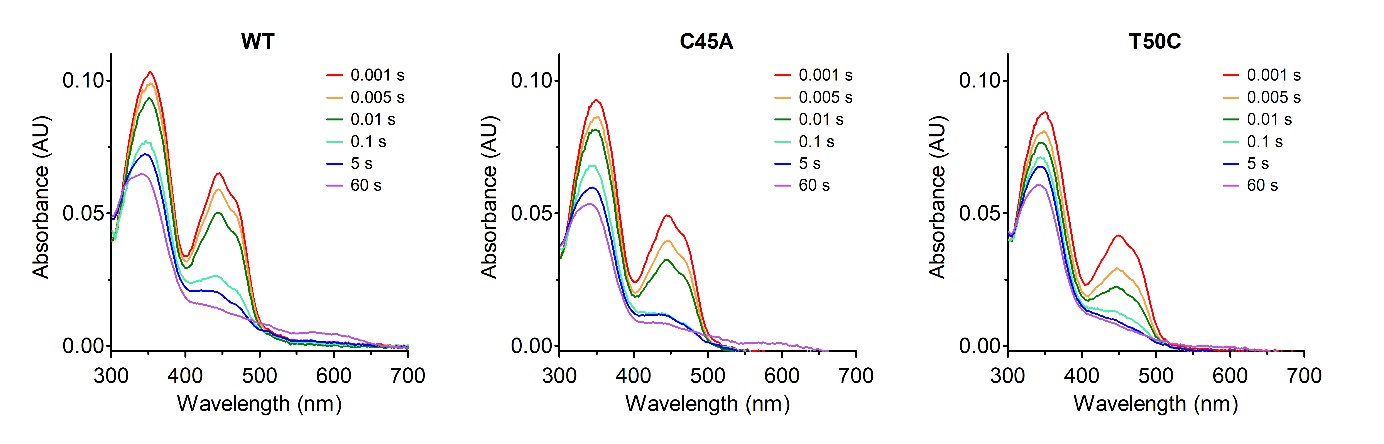
Supplemental Figure 9** Selected absorbance spectra of SthA variants upon anaerobic mixing with NADH. By mixing 50 µM NADH with SthA WT, T50C or C45A (7.5 µM) flavin reduction is observed (decrease of the absorbance peak at 450 nm) concomitant with the oxidation of the reduced nicotinamide cofactor (decrease of the absorbance peak at 340 nm). The data comes from biological duplicate (*n* = 2) using a single technical replicate for each dataset. Error bars are omitted for sake of clarity.


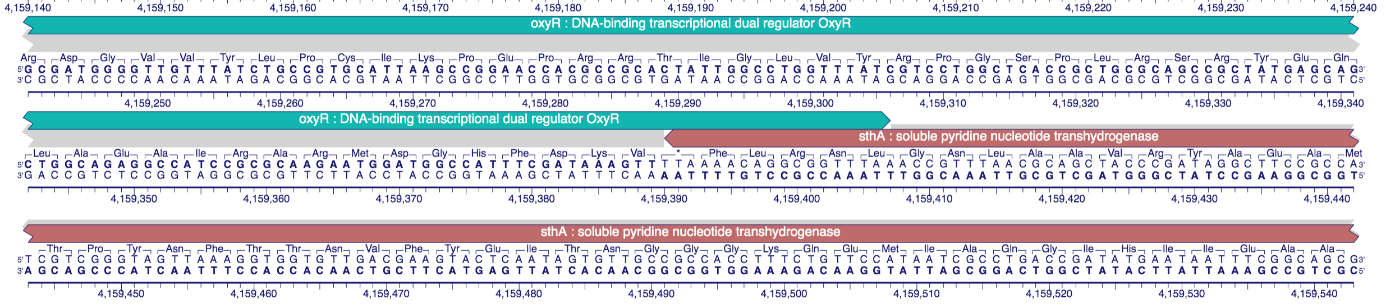


**Supplemental Figure 10** The overlap between the *udhA* and *oxyR* genes in *E. coli* K-12 genome. The 3 'end of the *udhA* gene shows an overlap of 12-nucleotides with the 3' end of *oxyR*. The protein product OxyR is a well-known regulator of the expression of antioxidant proteins in *E. coli*. The DNA sequence illustrates the nucleotides 4,159,140- 4,159,540 of the *Escherichia coli* K-12 substr. MG1655 reference genome available on the bioinformatics tool EcoCyc (https://ecocyc.org/).

**Supplementary Tables**

|  | **Activity (%)** | | | |
| --- | --- | --- | --- | --- |
| **Variant** | **No Adenine Nucleotide** | **+ 5.0 mM AMP** | **+ 5.0 mM ADP** | **+ 5.0 mM ATP** |
| WT | 100.0 ± 9.6 | 160.7 ± 2.9 | 171.1 ± 7.2 | 148.2 ± 8.9 |
| C45A | 100.0 ± 1.2 | 454.9 ± 19.0 | 991.8 ± 69.9 | 363.1 ± 24.9 |
| T50C | 100.0 ± 2.1 | 222.9 ± 12.9 | 319.2 ± 24.6 | 177.4 ± 16.0 |

**Supplemental Table 1** The percentual activity of WT, T50C and C45A is activated by ATP, ADP and AMP. Setting to 100% activity the initial velocity (first minute of reaction) of each specific variant in the absence of any adenine nucleotide, we calculated the increase of the activity in the presence of 5.0 mM AMP, ADP or ATP. The data comes from 3 independent repetitions (*n* = 3, the errors show the s.e.m.), while the reaction conditions are 50 mM Tris at pH 7.5, 1.0 mM NADH, 0.15 mM thioNADP^+^, 20 nM SthA. For clarity, 100% activity of WT corresponds to 8.66 ± 0.83 µmol min^-1^ mg^-1^; 100% activity of C45A corresponds to 0.55 ± 0.10 µmol min^-1^ mg^-1^; 100% activity of T50C corresponds to 0.92 ± 0.18 µmol min^-1^ mg^-1^.

| **Primers** | | **Sequence** |
| --- | --- | --- |
| **SthA-T50C** | Forward | 5’-ACCCACTGGGGCTGCATCCCGTCGAAA-3’ |
|  | Reverse | 5’-TTTCGACGGGATGCAGCCCCAGTGGGT-3’ |
| **SthA-C45A** | Forward | 5’-AATGTTGGCGGCGGTGCAACCCACTGG-3’ |
|  | Reverse | 5’-CCAGTGGGTTGCACCGCCGCCAACATT-3’ |

**Supplemental Table 2** The primers for the mutagenesis of the CXXXXT motif.

**Supplementary Experimental procedures**

**Negative staining and electron microscopy of SthA**

Freshly purified protein (around 20 µM) in SEC buffer was diluted to 1:100 with the same buffer and kept on ice. For the negative staining, 3 µL of the diluted protein were transferred to 10 µL of 2% uranyl acetate on a carbon-coated square 400 mesh grid (previously glow-discharged for 20 seconds at 5 mA on a ScanCoat Six (Edwards)) and let to adsorb for one minute. The removal of non-absorbed material was operated by blotting with filter paper. The grid screening was performed on a Philips CM120 electron microscope (Groningen Biomolecular Sciences and Biotechnology Institute, the Netherlands) equipped with a Gatan UltraScan 4000SP CCD Camera. The images were acquired under low-dose conditions, while their brightness and contrast were adjusted on ImageJ.
